# Effect of Medical Marijuana Card Ownership on Pain, Insomnia, and Affective Disorder Symptoms in Adults: A Randomized Clinical Trial

**DOI:** 10.1001/jamanetworkopen.2022.2106

**Published:** 2022-03-18

**Authors:** Jodi M. Gilman, Randi M. Schuster, Kevin W. Potter, William Schmitt, Grace Wheeler, Gladys N. Pachas, Sarah Hickey, Megan E. Cooke, Alyson Dechert, Rachel Plummer, Brenden Tervo-Clemmens, David A. Schoenfeld, A. Eden Evins

**Affiliations:** 1 Department of Psychiatry, Massachusetts General Hospital (MGH), Boston; 2 Department of Psychiatry, Harvard Medical School, Boston, Massachusetts; 3 Athinoula A. Martinos Center for Biomedical Imaging, Department of Radiology, MGH, Harvard Medical School, Charlestown; 4 Department of Biostatistics, MGH, Boston

## Abstract

**Importance:**

Despite the legalization and widespread use of cannabis products for a variety of medical concerns in the US, there is not yet a strong clinical literature to support such use. The risks and benefits of obtaining a medical marijuana card for common clinical outcomes are largely unknown.

**Objective:**

To evaluate the effect of obtaining a medical marijuana card on target clinical and cannabis use disorder (CUD) symptoms in adults with a chief concern of chronic pain, insomnia, or anxiety or depressive symptoms.

**Design, Setting, and Participants:**

This pragmatic, single-site, single-blind randomized clinical trial was conducted in the Greater Boston area from July 1, 2017, to July 31, 2020. Participants were adults aged 18 to 65 years with a chief concern of pain, insomnia, or anxiety or depressive symptoms. Participants were randomized 2:1 to either the immediate card acquisition group (n = 105) or the delayed card acquisition group (n = 81). Randomization was stratified by chief concern, age, and sex. The statistical analysis followed an evaluable population approach.

**Interventions:**

The immediate card acquisition group was allowed to obtain a medical marijuana card immediately after randomization. The delayed card acquisition group was asked to wait 12 weeks before obtaining a medical marijuana card. All participants could choose cannabis products from a dispensary, the dose, and the frequency of use. Participants could continue their usual medical or psychiatric care.

**Main Outcomes and Measures:**

Primary outcomes were changes in CUD symptoms, anxiety and depressive symptoms, pain severity, and insomnia symptoms during the trial. A logistic regression model was used to estimate the odds ratio (OR) for CUD diagnosis, and linear models were used for continuous outcomes to estimate the mean difference (MD) in symptom scores.

**Results:**

A total of 186 participants (mean [SD] age 37.2 [14.4] years; 122 women [65.6%]) were randomized and included in the analyses. Compared with the delayed card acquisition group, the immediate card acquisition group had more CUD symptoms (MD, 0.28; 95% CI, 0.15-0.40; *P* < .001); fewer self-rated insomnia symptoms (MD, –2.90; 95% CI, –4.31 to –1.51; *P* < .001); and reported no significant changes in pain severity or anxiety or depressive symptoms. Participants in the immediate card acquisition group also had a higher incidence of CUD during the intervention (17.1% [n = 18] in the immediate card acquisition group vs 8.6% [n = 7] in the delayed card acquisition group; adjusted odds ratio, 2.88; 95% CI, 1.17-7.07; *P* = .02), particularly those with a chief concern of anxiety or depressive symptoms.

**Conclusions and Relevance:**

This randomized clinical trial found that immediate acquisition of a medical marijuana card led to a higher incidence and severity of CUD; resulted in no significant improvement in pain, anxiety, or depressive symptoms; and improved self-rating of insomnia symptoms. Further investigation of the benefits of medical marijuana card ownership for insomnia and the risk of CUD are needed, particularly for individuals with anxiety or depressive symptoms.

**Trial Registration:**

ClinicalTrials.gov Identifier: NCT03224468

## Key Points

**Question:**

What are the risks and benefits of obtaining a medical marijuana card for adults who seek medical marijuana for pain, insomnia, and anxiety or depressive symptoms?

**Findings:**

In this randomized clinical trial involving 186 participants, immediate acquisition of a medical marijuana card increased the incidence and severity of cannabis use disorder (CUD) and resulted in no significant improvement in pain, anxiety, or depressive symptoms, but improved self-reported sleep quality.

**Meaning:**

Findings from this study suggest the need for further investigation into the benefits of medical marijuana card ownership for insomnia symptoms and the risk of CUD, particularly for those with anxiety or depressive symptoms.

## Teaser

This randomized clinical trial examines the risks and benefits of a medical marijuana card, including the development of cannabis use disorder and improvement of insomnia symptoms.

## Introduction

Despite inconclusive evidence of its efficacy^1^ and little information on its risk, medical cannabis has surged in popularity. As of December 2021, approximately 12 countries, including Canada, the United Kingdom, and Australia, and 36 US states and the District of Columbia have commercialized cannabis for medical use, making it accessible through the use of a medical marijuana card for myriad health conditions. Given the increasing prevalence of cannabis use for medical concerns, well-designed studies are needed to assess the effect of cannabis product use on target symptoms and associated adverse medical and psychiatric events, particularly the development of cannabis use disorder (CUD).

Cannabis has been reported to improve pain, sleep, and anxiety and depressive symptoms^2^ and is commonly sought for these concerns.^3^ However, according to national data, 3 in 10 US adults who use cannabis develop CUD, with 23% developing severe CUD^4^ and often with a tolerance to delta-9-tetrahydrocannabinol (THC) and withdrawal symptoms.^5,6^ Data are lacking on whether the rates of addiction in adults with a medical marijuana card are similar to the rates in those who use cannabis for recreational purposes. In addition, cannabis use has been associated with psychotic and depressive disorders, mania, suicide, and cognitive impairment.^2,7,8,9,10^ Thus, it is imperative to better understand both the benefits and potential risks of cannabis use for medical concerns in the current regulatory environment.

We conducted a randomized clinical trial (RCT) to evaluate the effect of obtaining a medical marijuana card on target clinical and CUD symptoms in adults with a chief concern of chronic pain, insomnia, or anxiety or depressive symptoms. Participants were randomized to acquire a medical marijuana card immediately or to be placed on a waiting list to procure a card. We hypothesized modest improvements in pain and insomnia symptoms along with worsened CUD and depressive symptoms over 12 weeks in participants in the immediate card acquisition group.

## Methods

This pragmatic, single-site, single-blind RCT was conducted in the Greater Boston area from July 1, 2017, to July 31, 2020, approximately 2 years after medical cannabis dispensaries began operating in Massachusetts. Study procedures (Supplement 1) were approved by the Mass General Brigham Human Research Committee. Written informed consent was obtained from all study participants, and they received financial compensation for participation. We followed the Consolidated Standards of Reporting Trials (CONSORT) reporting guideline.^11^

Participants were recruited from clinical sites (eg, local Massachusetts General Hospital clinics) and from the community. Eligible participants were aged 18 to 65 years who sought medical marijuana to improve pain, insomnia, and anxiety or depressive symptoms. Daily cannabis use, CUD diagnosis at screening or baseline, cancer, psychosis, and current substance use disorders (except for mild or moderate alcohol use disorder and nicotine use disorder) were the criteria for exclusion. The eMethods in Supplement 2 provides detailed inclusion criteria.

### Randomization and Masking

**Figure 1.**
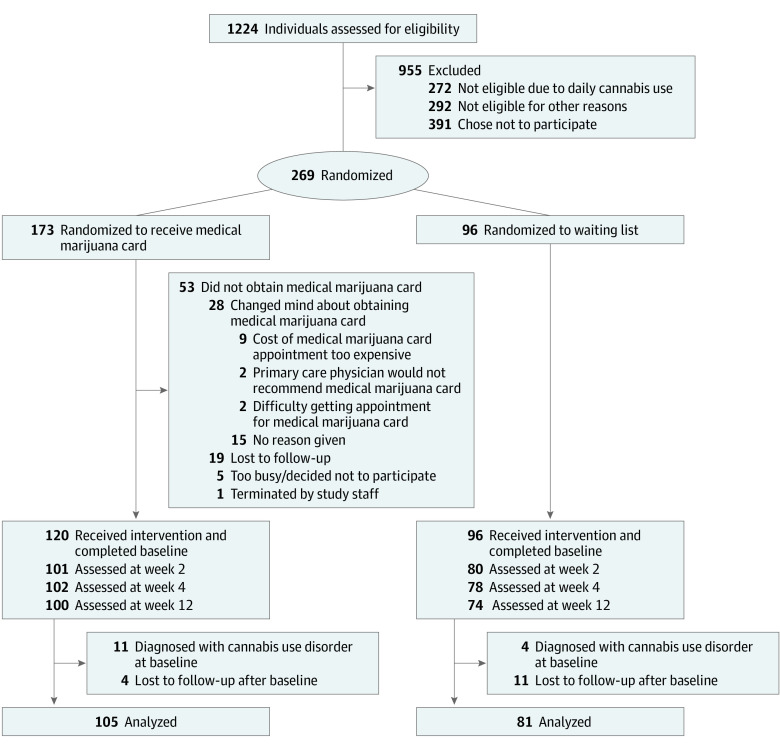
Study Flow Diagram

Participants were randomized to either the immediate card acquisition group or to the delayed card acquisition group (Figure 1). In the immediate card acquisition group, participants were allowed to obtain a card immediately. In the delayed card acquisition group, participants were asked to wait 12 weeks to obtain a medical marijuana card. Participants from both groups could choose their cannabis products, dose, and frequency of use, thus allowing for a pragmatic evaluation of the effect of a medical marijuana card within the system in place for physician recommendation and product regulation and distribution.

Randomization was stratified by sex, age (18-25 vs 26-65 years), and primary medical concern (pain, insomnia, or anxiety or depression). Because we expected that financial and logistical constraints might limit the procurement of a medical marijuana card, increasing the dropout rate in the immediate card acquisition group between randomization and the start of study procedures, we randomized participants 2:1 (immediate card acquisition group to delayed card acquisition group) in each stratum to generate groups that were approximately equal in size for analysis. Randomization was accomplished using a computer-generated random number sequence, which was created and executed by an independent statistician.

Blinding of participants after group randomization was not possible because of the study design. Rater blinding is discussed in the eMethods in Supplement 2.

### Procedures and Measures

Sociodemographic information, medical history, and psychiatric diagnoses were collected at screening, before randomization, using the Mini International Neuropsychiatric Interview^12^ (Table 1). Participants in the immediate card acquisition group then obtained their cards, and all participants returned for in-person visits at baseline and at weeks 2, 4, and 12 after randomization as well as participated in a telephone visit at week 8 for an assessment of adverse events (AEs). Participants in the immediate card acquisition group were responsible for arranging for and paying the costs of obtaining a medical marijuana card and cannabis products. The trial did not provide or pay for the medical marijuana cards or cannabis products used by the participants.

**Table 1.** Participant Characteristics by Randomization Group

Variable	No. (%)		
	All participants (N = 186)	Immediate card acquisition group (n = 105)	Delayed card acquisition group (n = 81)
Age, mean (SD), y	37.2 (14.4)	37.9 (14.3)	36.3 (14.5)
Sex			
Female	122 (65.6)	72 (68.6)	50 (61.7)
Male	64 (34.4)	33 (31.4)	31 (38.3)
Race and ethnicity^a^			
African American or Black	14 (7.5)	7 (6.7)	7 (8.6)
Asian	10 (5.4)	6 (5.7)	4 (4.9)
Hispanic	11 (5.9)	4 (3.8)	7 (8.6)
Multiracial	6 (3.2)	3 (2.9)	3 (3.7)
Pacific Islander	0	0	0
White	152 (81.7)	88 (83.8)	64 (79.0)
Unknown^b^	4 (2.2)	1 (1.0)	3 (3.7)
Educational level			
High school diploma	10 (5.4)	4 (3.8)	6 (7.4)
Some college	36 (19.4)	16 (15.2)	20 (24.7)
College degree			
2-y	4 (2.2)	4 (3.8)	0
4-y	61 (32.8)	35 (33.3)	26 (32.1)
Some graduate school	73 (39.2)	46 (43.8)	27 (33.3)
Years of education, mean (SD)	16.5 (2.5)	16.6 (2.3)	16.3 (2.7)
Cannabis use frequency ≥weekly	52 (28.0)	23 (21.9)	29 (35.8)
Primary concern^c^			
Pain	61 (32.8)	37 (35.2)	24 (29.6)
Insomnia	42 (22.6)	22 (21.0)	20 (24.7)
Anxiety or depression	83 (44.6)	46 (43.8)	37 (45.7)

^a^ Participants self-reported their race and ethnicity.

^b^ Unknown included missing race and ethnicity information.

^c^ Primary concern was defined by participant self-report of the condition for which they were seeking medical cannabis.

Verification of compliance within the randomization group was self-reported by participants. All participants in the immediate card acquisition group reported obtaining a card before the baseline visit, and all participants in the delayed card acquisition group agreed to wait 12 weeks to procure a card. Quantity and frequency of cannabis use; sleep quality; and depression, anxiety, and pain symptoms were reported and assessed at every visit via interviews and daily via smartphone diaries. Participants could continue their ongoing medical or psychiatric care during the trial.

### Primary, Secondary, and Exploratory Outcomes

Outcomes were assessed at baseline and at weeks 2, 4, and 12 except for the cognitive and the Short-Form Health Survey (SF-12) measures,^13^ which were not assessed at week 2. The 5 primary outcomes were (1) CUD symptoms, which were assessed by doctorate-level (J.M.G., R.M.S., and M.E.C.) or registered nurse–level (S.H.) investigators using the CUD Checklist for the *Diagnostic and Statistical Manual of Mental Disorders (Fifth Edition)* (score range: 0-11, with higher scores indicating more severe CUD)^14^; (2 and 3) anxiety and depressive symptoms, which were assessed separately using the Hospital Anxiety and Depression Scale (score range: 0-21, with 0-7 indicating normal, 8-10 indicating borderline abnormal [borderline anxiety or depression], and 11-21 indicating abnormal levels)^15^; (4) pain severity, which was assessed using the severity subscale of the Brief Pain Inventory (score range: 0-10, with 10 being the worst imaginable pain)^16^; and (5) insomnia symptoms, which were assessed using the Athens Insomnia Scale (score range: 0-24, with higher scores indicating more severe sleep difficulties).^17^

Secondary outcomes included physical and mental (assessed using the SF-12 Physical and Mental scales; score range: 0-100; using T-scores, higher scores indicate better physical health and mental health functioning)^13^ as well as cognitive (assessed using the Cambridge Neuropsychological Test Automated Battery [CANTAB]) measures.^18^ CANTAB tasks included the Attention Switching Task (attention shifting or executive function), Rapid Visual Information Processing (sustained attention), Paired Associates Learning (visual memory), Spatial Working Memory (spatial working memory or executive function), and Verbal Recognition Memory (verbal memory). Alternative forms of CANTAB tasks were administered when available to minimize practice effects. The eMethods in Supplement 2 provides descriptions of all CANTAB tasks.

Exploratory outcomes were as follows: cannabis misuse (assessed with the Cannabis Use Disorders Identification Test [CUDIT]; score range: 0-32, with higher scores indicating more problematic cannabis use, ≥8 indicating hazardous cannabis use, and ≥12 indicating a possible CUD),^19^ marijuana craving (assessed with the Marijuana Craving Questionnaire; score range: 12-84, with higher scores indicating greater or more severe marijuana craving),^20^ pain interference (assessed with the Brief Pain Inventory Pain Interference scale; score range: 0-10, with higher scores indicating worse pain interference), pain catastrophizing (assessed with the Pain Catastrophizing Scale; score range: 0-52, with higher scores indicating greater pain catastrophizing),^21^ perceived stress (assessed with the Perceived Stress Scale; score range: 0-40, with higher scores indicating greater perceived stress),^22^ suicidal thoughts (assessed with the Concise Health Risk Tracking scale; score range: 12-60, with higher scores indicating more suicidal thoughts),^23^ and illness severity and improvement (assessed with the Clinical Global Impression [CGI]^14^ Severity subscale [score range: 1-7, with the highest scores indicating greatest severity of illness] and the Improvement subscale [score range: –3 to 3, with higher scores indicating worse-than-baseline condition and negative scores indicating improvement]).^24^ Cannabis use at each visit was assessed on a 7-point Likert scale (score range: 1-7, with higher scores indicating greater frequency of use).

### Urinalysis and Adverse Events

Urine samples were collected from participants at each study visit and then shipped on dry ice to the University of Colorado Department of Anesthesiology, where the samples were analyzed for cannabinoids using high-performance liquid chromatography with tandem mass spectrometry.^25^ This assay quantified THC, cannabidiol (CBD), primary metabolites, and 15 other cannabinoids.^26^

We identified AEs via an open-ended question: “Since the last time we saw/spoke to you, have you experienced any medical events such as illness or injury, or worsening symptoms?” Participants who reported any substantial worsening of their psychiatric condition underwent a psychiatric evaluation by a mental health professional. Cannabis use disorder symptoms were a primary outcome and assessed by our doctorate-level (J.M.G., R.M.S., and M.E.C.) or nursing-level (S.H.) study staff, who recommended CUD treatment referral and reduced cannabis use for participants who developed moderate or severe CUD.

### Sample Size and Power

We aimed to recruit 200 participants. To identify a difference in onset of CUD symptoms in the immediate card acquisition group vs the delayed card acquisition group, assuming that the mean number of symptoms was 0.4 (corresponding to 20% of participants developing CUD) in the immediate card acquisition group vs 0.1 in the delayed card acquisition group (corresponding to 5% of participants developing CUD) at a 2-sided α = .05 significance level, we estimated a power of 85%. To identify differences in pain, insomnia, and anxiety or depressive symptoms, assuming that a clinically significant effect would be a 30% reduction in the presenting medical symptom in the immediate card acquisition group and a 5% reduction in the delayed card acquisition group at a 2-sided α = .05 significance level based on effect sizes in the literature,^27,28,29^ we estimated a power of 84% for pain, 90% for insomnia, and 84% for anxiety or depressive symptoms, with sample sizes of approximately 33 in each subgroup. The eMethods in Supplement 2 include the calculation of sample size and power.

### Statistical Analysis

All participants who completed a baseline assessment and at least 1 postbaseline visit were included in the analysis. We used an evaluable population approach for the statistical analyses that included all participants in the delayed card acquisition group and all participants in the immediate card acquisition group who obtained a card and had 1 postbaseline assessment. Analyses used generalized estimating equations to account for repeated measures for a given individual across the primary, secondary, and exploratory outcomes. The statistical model assumed a constant effect of a medical marijuana card over time (weeks 2, 4, and 12 visits), with baseline scores as a covariate. A logistic regression model was used to estimate the odds ratio (OR) for CUD diagnosis, and linear models were used for continuous outcomes to estimate the mean difference (MD) in symptom scores. All tests and CIs were 2 sided, and statistical significance was defined as a *P* ≤ . 05 for the primary outcomes. We reported original^30^ and adjusted *P* values that were corrected for multiple comparisons.^31^ Results of secondary or exploratory analyses were reported as point estimates with 95% CIs. Analyses were performed using R, version 4.0.2 (R Foundation for Statistical Computing).

We conducted 2 post hoc sensitivity analyses. First, owing to a substantial number of dropouts in the immediate card acquisition group (participants who did not obtain a card; 53 of 173 [30.6%]), we ran a multivariable logistic regression to test whether baseline characteristics (eg, age, sex, race and ethnicity [which were self-reported and included African American or Black; Asian; Hispanic; multiracial; Pacific Islander; White; or unknown, including missing race and ethnicity information], educational level, baseline level of cannabis use, primary concern, and symptom severity) were different in the immediate card acquisition group among participants who obtained a card vs those who did not. To test whether the probability of obtaining a card biased participants in both randomization groups, we calculated propensity scores for all participants using the logistic regression model (model run in the immediate card acquisition group and applied to both groups). We entered these weightings in the analyses of primary outcomes using generalized estimating equations by the inverse of this propensity score.

Second, because all participants could choose a variety of cannabis products with input from licensed medical marijuana dispensaries or elsewhere, we conducted a modified per protocol sensitivity analysis that compared the primary outcomes in participants in the immediate card acquisition group whose urine samples at week 12 (end of the intervention) had detectable levels of THC, CBD, or metabolites with those of participants in the delayed card acquisition group without detectable levels of THC, CBD, or metabolites at week 12.

## Results

Of the 1224 individuals who were screened via telephone, 269 were enrolled and randomized and 186 (mean [SD] age 37.2 [14.4] years; 122 women [65.6%] and 64 men [34.4%]) completed baseline and at least 1 postbaseline visits and were included in the analyses (immediate card acquisition group: n = 105; delayed card acquisition group: n = 81). Table 1 and eTables 1 and 2 in Supplement 2 provide the characteristics of the participants.

### Outcomes

**Figure 2.**
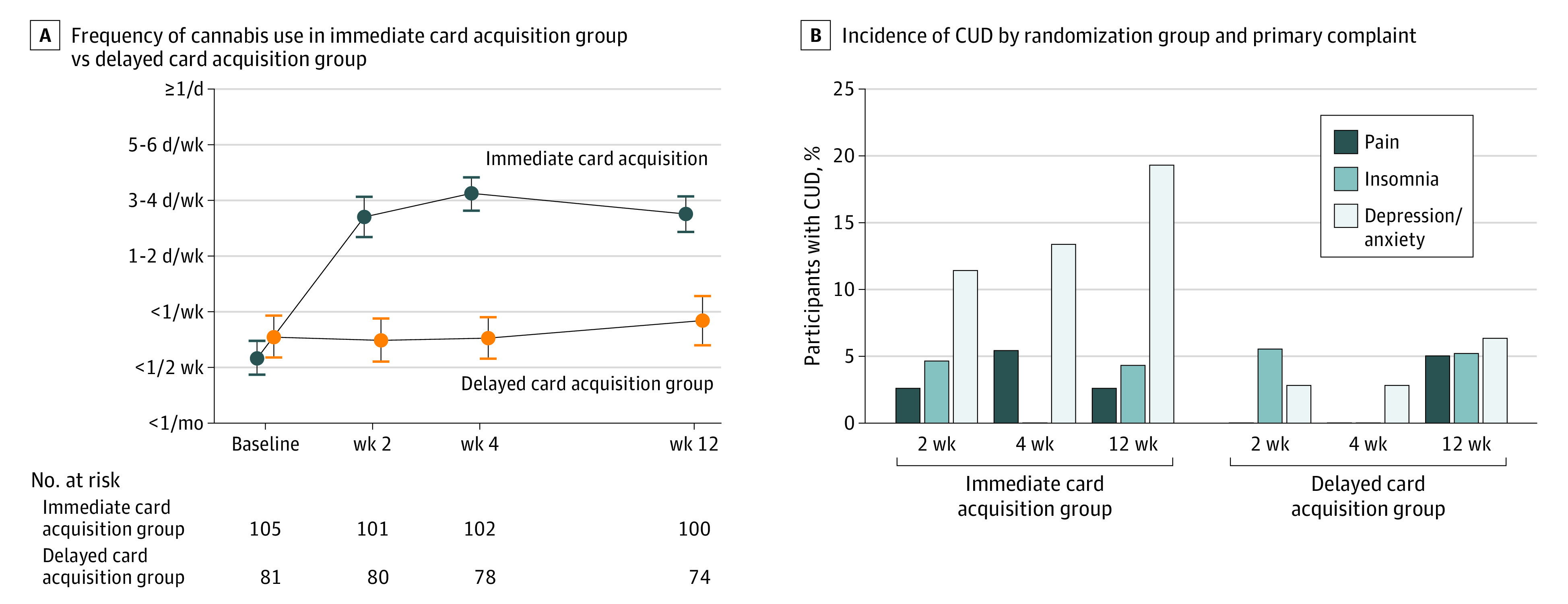
Frequency of Cannabis Use and Incidence of Cannabis Use Disorder (CUD) Diagnoses in Immediate vs Delayed Card Acquisition Groups A, Cannabis use was assessed via a self-reported scale, which asked for frequency of cannabis use at each visit. There was a significant increase in use in the immediate card acquisition group vs the delayed card acquisition group (2.44; 95% CI, 2.08-2.81; *P* < .001). B, Cannabis use disorder was defined as 2 or more CUD symptoms on an 11-point scale. The odds of developing CUD were 2.9-fold higher in the immediate card acquisition group vs the delayed card acquisition group (adjusted odds ratio, 2.88; 95% CI, 1.17-7.07; *P* = .02).

**Figure 3.**
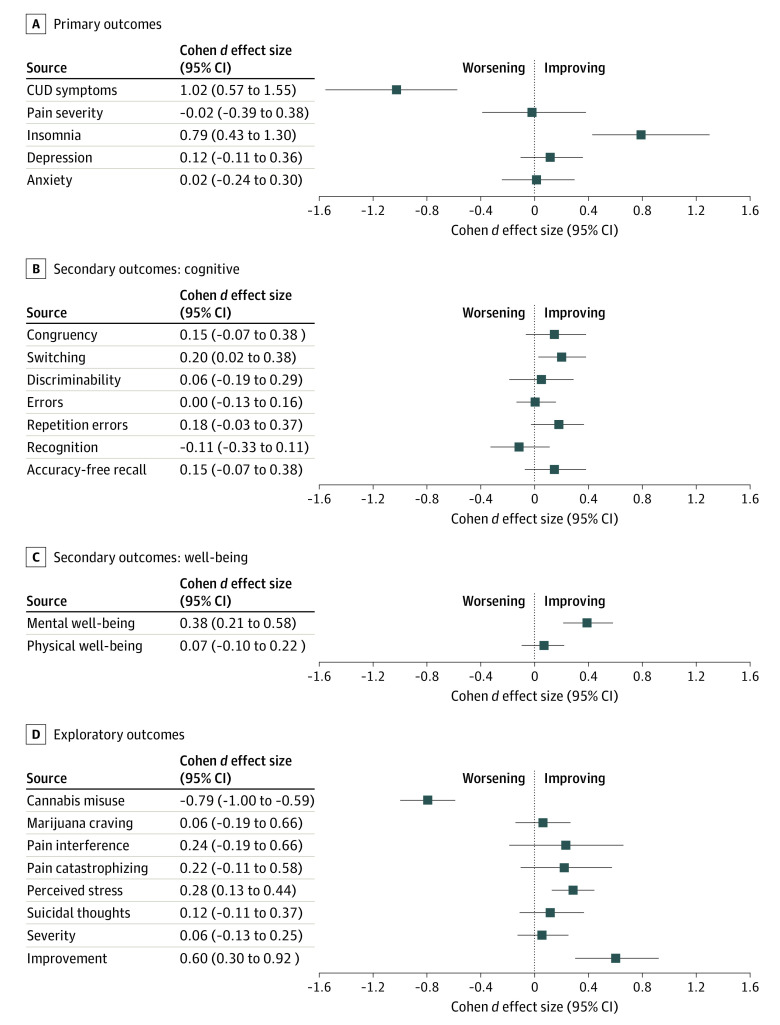
Effect Sizes for Primary, Secondary, and Exploratory Outcomes Cohen *d* and 95% CIs were obtained from generalized estimating equations linear models. For clarity, all outcomes are plotted on the x-axis by worsening or improving, rather than by item score.

As expected, the immediate card acquisition group reported significantly greater cannabis use throughout the intervention period than the delayed card acquisition group (likert scale difference: 2.44; 95% CI, 2.08-2.81; *P* < .001) (Figure 2A and eFigure in Supplement 2). Participants in the immediate card acquisition group reported a greater number of CUD symptoms over the 12-week trial than those in the delayed card acquisition group (MD, 0.28; 95% CI, 0.15-0.40; *P* < .001) (Table 2 and Figure 3; eFigure in Supplement 2). Participants in the immediate card acquisition group had reduced self-rated insomnia symptoms over the 12-week intervention compared with those in the delayed card acquisition group (MD, –2.90; 95% CI, –4.31 to –1.51; *P* < .001). There was no significant group effect on pain, anxiety, or depressive symptom ratings (Table 2 and Figure 3). eTables 3 and 4 in Supplement 2 show the self-reported methods of use and urine cannabinoid metabolite measurements in each group.

**Table 2.** Primary Outcomes by Randomization Group

Outcome	Visit	Immediate card acquisition group		Delayed card acquisition group		Mean difference (95% CI)^a^	Cohen *d* (95% CI)	*P* value^a^	Adjusted *P* value^a^
		No. of participants	Mean (SD) score	No. of participants	Mean (SD) score				
CUD symptoms^b^	Baseline	105	0.08 (0.27)	81	0.09 (0.28)	0.28 (0.15 to 0.40)	1.02 (0.57 to 1.55)	<.001	<.001
	Wk 2	101	0.30 (0.67)	80	0.16 (0.56)				
	Wk 4	102	0.33 (0.68)	78	0.05 (0.36)				
	Wk 12	100	0.55 (0.95)	74	0.16 (0.50)				
Pain severity^c^	Baseline	37	2.8 (2.3)	24	3.9 (2.4)	0 (–0.8 to 0.9)	0.02 (–0.38 to 0.39)	.93	.93
	Wk 2	37	3.2 (2.2)	24	3.6 (2.4)				
	Wk 4	36	2.4 (2.3)	22	3.1 (2.6)				
	Wk 12	37	2.5 (2.4)	21	3.1 (2.6)				
Insomnia symptoms^d^	Baseline	22	12.4 (4.4)	20	12.2 (2.7)	–2.90 (–4.31 to –1.51)	–0.79 (–1.30 to –0.43)	<.001	<.001
	Wk 2	21	10.0 (5.4)	19	11.6 (3.7)				
	Wk 4	22	8.8 (3.4)	20	12.1 (2.6)				
	Wk 12	22	7.6 (4.9)	20	11.2 (4.7)				
Depressive symptoms^e^	Baseline	46	6.1 (3.7)	37	5.2 (4.3)	–0.5 (–1.4 to 0.4)	–0.12 (–0.36 to 0.11)	.30	.50
	Wk 2	43	6.0 (4.5)	37	4.4 (3.6)				
	Wk 4	44	5.2 (4.1)	36	5.2 (3.9)				
	Wk 12	41	4.9 (4.1)	33	5.5 (4.2)				
Anxiety symptoms^e^	Baseline	46	9.4 (4.4)	37	9.4 (4.1)	–0.1 (–1.1 to 1.0)	–0.02 (–0.30 to 0.24)	.90	.93
	Wk 2	43	8.3 (4.3)	37	8.3 (3.8)				
	Wk 4	44	8.5 (4.5)	36	8.4 (4.1)				
	Wk 12	41	8.3 (4.4)	33	8.4 (3.7)				

Abbreviation: CUD, cannabis use disorder.

^a^ Estimated raw and adjusted differences and associated *P* values were based on a generalized estimating equation linear model. Adjustments to *P* values for multiple comparisons were based on Benjamini and Hochberg.^31^

^b^ CUD symptoms were assessed with the CUD Checklist for *Diagnostic and Statistical Manual of Mental Disorders (Fifth Edition)* (score range: 0-11, with higher scores indicating more severe CUD). This measure was analyzed in all participants.

^c^ Pain severity was assessed with the severity subscale of the Brief Pain Inventory (score range: 0-10, with 10 being the worst imaginable pain). This measure was analyzed only in participants with a primary concern of pain.

^d^ Insomnia symptoms were assessed with the Athens Insomnia Scale (score range: 0-24, with higher scores indicating more severe sleep difficulties). This measure was analyzed only in participants with a primary concern of insomnia.

^e^ Depression and anxiety symptoms were assessed with the Hospital Anxiety and Depression Scale (score range: 0-21, with 0-7 indicating normal, 8-10 indicating borderline abnormal [borderline anxiety or depression], and 11-21 indicating abnormal levels). This measure was analyzed only in participants with a primary concern of depression or anxiety.

The immediate card acquisition group had greater score improvement in mental well-being on the SF-12 (MD, 4.67; 95% CI, 2.63-6.71; Cohen *d* = 0.39) but showed no significant effect on physical well-being compared with the delayed card acquisition group. There were no significant group effects on cognitive task performance (all with Cohen *d *≤0.2) (eTable 5 in Supplement 2; Figure 3).

Participants in the immediate card acquisition group were more likely to develop a *Diagnostic and Statistical Manual of Mental Disorders (Fifth Edition)*–based CUD diagnosis over the trial period vs those in the delayed card acquisition group (adjusted OR, 2.88; 95% CI, 1.17-7.07; *P* = .02). Throughout the 12 weeks, 18 participants (17.1%) in the immediate card acquisition group had a CUD diagnosis during at least 1 time point vs 7 participants (8.6%) in the delayed card acquisition group. Within the anxiety or depressive symptom subgroup, throughout the 12 weeks, 13 of 46 participants (28.3%) in the immediate card acquisition group and 4 of 37 participants (10.8%) in the delayed card acquisition group developed CUD (Figure 2B). Most CUD diagnoses were mild (eTable 6 in Supplement 2). eTable 7 in Supplement 2 includes a count of the CUD symptoms that were endorsed within each group. The immediate card acquisition group reported greater CUD symptom severity on the CUDIT than the delayed card acquisition group, with a large effect size (CUDIT score MD, 2.22; 95% CI, 1.65-2.78; Cohen *d* = 0.8) (eTable 8 in Supplement 2).

The immediate card acquisition group had higher scores on the CGI-Improvement subscale, with a medium effect size (MD, 0.37; 95% CI, 0.56-0.19; Cohen *d* = 0.6) and no notable difference from the delayed card acquisition group in scores on the CGI-Severity subscale. The immediate card acquisition group also had lower Perceived Stress Scale scores (MD, –2.09; 95% CI, –3.19 to –0.99; Cohen *d* = –0.3). There were no group effects on other exploratory outcomes (Figure 3; eTable 8 in Supplement 2).

### Sensitivity Analyses and Adverse Events

There were no significant factors that indicated which participants in the immediate card acquisition group would obtain a card (n = 120) vs which participants would not (n = 53) (eTable 9 in Supplement 2), and weighting the primary outcomes by propensity scores to calculate who would obtain a card yielded nearly identical estimates with no change in inferences (eTable 10 in Supplement 2). Comparing the primary outcomes in participants in the immediate card acquisition group with detectable levels of THC, CBD, or metabolites in urine at week 12 vs participants in the delayed card acquisition group without detectable levels of THC, CBD, or metabolites also yielded results that were similar to those presented in the main analyses (eTable 11 in Supplement 2). Sensitivity analyses that included only assessments with confirmed blinded raters also yielded similar results (eTable 12 in Supplement 2).

One or more AEs were reported by 85 of 105 participants (80.1%) in the immediate card acquisition group and by 60 of 81 participants (74.1%) in the delayed card acquisition group (eTables 13-14 in Supplement 2). A serious AE (cardiac event) occurred in 1 participant in the immediate card acquisition group.

## Discussion

In this single-blind, pragmatic RCT involving people who sought cannabis products to improve insomnia, pain, or anxiety or depressive symptoms, participants in the immediate card acquisition group developed a greater number of CUD symptoms and had a higher incidence and greater severity of CUD diagnosis over the 12-week trial after obtaining a card compared with those in the delayed card acquisition group. Analyzed by chief concerns of pain, insomnia, and anxiety or depressive symptoms, participants in the immediate card acquisition group reported improved insomnia but no significant changes in pain severity and anxiety or depressive symptoms.

This study indicated that obtaining a medical marijuana card and using cannabis products from a dispensary, with the required medical oversight, for medical concerns of pain, anxiety, or depressive symptoms increased the risk for developing CUD without significantly improving symptoms. The odds of developing CUD were almost 2.9-fold higher in the immediate card acquisition group than in the delayed card acquisition group. Epidemiologic surveys of recreational cannabis use found that 3 in 10 adults who use cannabis develop CUD,^4^ but it is unknown whether adults with a medical marijuana card would develop CUD at a lower rate than recreational users. In this trial, 17.1% of participants in the immediate card acquisition group developed a CUD diagnosis throughout the 12 weeks of study after acquiring a card. Although this incidence is lower than the 12-month incidence of CUD reported by Hasin et al,^4^ the current study assessed the onset of CUD after only 12 weeks of medical marijuana card ownership. Thus, further research is warranted with a longer follow-up of people who obtain a card to better understand CUD risk in this group. This trial showed that CUD can develop at a fast rate within the first 12 weeks of medical marijuana card ownership, suggesting that those with a card may develop CUD at a similar rate as those who use cannabis recreationally and that the motive for use (eg, medical) may not be protective. Although most cases of CUD onset in the trial were mild, with 2 to 4 symptoms, these symptoms developed over a short, 12-week initial exposure. The most commonly reported CUD symptoms were higher tolerance and continued use despite the recurrent physical or psychological problems caused or exacerbated by cannabis.

Most of those who developed CUD sought a medical marijuana card for affective symptoms; 28.3% of participants in the immediate card acquisition group with a chief concern of anxiety or depression and 10.8% of participants in the delayed card acquisition group experienced CUD onset during the 12-week study. Thus, consistent with findings from epidemiologic studies,^32,33^ people with affective symptoms who have access to cannabis through medical marijuana cards may be particularly at risk of CUD onset. Cannabis use disorder frequently co-occurs with affective disorders, particularly depression.^34^ Individuals with affective disorders have 3.9-fold (95% CI, 2.8-5.3) higher odds of meeting CUD diagnostic criteria,^35^ and bidirectional associations between cannabis use and depression have been reported.^32,33^ These data suggest that a medical marijuana card may pose a high risk or may even be contraindicated for people with affective disorders. This finding is important to replicate because depression has been reported as the third most common reason that people seek a medical marijuana card.^3^

The finding of improved self-reported insomnia with a large effect size merits further study. The endocannabinoid system has been described as critical in regulation of the circadian sleep-wake cycle,^36,37^ including maintenance and promotion of sleep.^38^ Although RCTs of cannabis for primary insomnia are lacking, small effects of cannabinoids on secondary sleep outcomes have been reported.^2^ Those with a primary insomnia concern were unlikely to develop CUD, suggesting a potential clinical utility of cannabinoids for insomnia. Thus, further study of the effect of cannabinoids on people with primary insomnia is warranted, using objective measures and self-assessments of sleep, along with analysis of CUD symptoms over a period longer than this 12-week trial.

Although pain, anxiety, and depressive symptoms were the common reasons cited for the use of cannabinoids, we detected no substantial benefit of a medical marijuana card for any of these outcomes. This null finding of medical marijuana card ownership on pain severity, pain interference, and pain catastrophizing is consistent with a recent systematic review and expert consensus recommendation against the use of any cannabinoids for chronic pain.^39^ Because preclinical studies have suggested that the endocannabinoid system, through CB1 and CB2 receptors, is a key regulator of pain sensation, these receptors remain potential targets for pain therapeutics.^39,40,41^ As such, mechanistic pain trials are warranted. We did observe an effect of medical marijuana card ownership on mental well-being and perceived stress that may be relevant to these health concerns. This finding deserves follow-up, and the potential risk of CUD should be weighed against the potential benefit to perceived stress and well-being. No AEs related to psychotic symptoms, mania, hypomania, or suicidal ideation or behavior were observed in the immediate card acquisition group during the trial.

The findings of this trial may provide insight into the potential risks and benefits of a medical marijuana card for people seeking cannabis for medical concerns. Although the development of CUD may suggest the likelihood of future medical marijuana card ownership,^42^ participants in this study did not have evidence of problematic cannabis use at baseline, and yet some participants developed CUD over the first 12 weeks of card ownership. Thus, clinicians and patients are advised to consider the risks of cannabis use, especially in those with affective disorders, who may be particularly susceptible to developing CUD. In many US states with medical marijuana laws, cannabis is approved for a variety of medical conditions, but little evidence of its efficacy is available. Thus, further study is needed to replicate and extend the findings of this trial, specifically the risks and benefits of cannabis use for specific medical conditions and the rate of CUD development over longer periods and under different health conditions, particularly mental health conditions. Such work can inform clinical decision-making on whether to initiate cannabis for specific medical concerns.

### Limitations

This study has several limitations. We assessed the risks and benefits of medical marijuana card ownership among participants who chose from a variety of cannabis products at self-titrated doses and who were aided by community physicians who recommended cannabis products for pain, insomnia, anxiety, or depressive symptoms. Therefore, we cannot comment on the pharmacological effects and risks or benefits of specific cannabinoids at specific doses for the health concerns we examined. Determination of the cannabinoid doses used is challenging in this circumstance.^26^ We followed expert consensus guidelines^43^ by using timeline follow-back methods to document the frequency of use, rather than quantity or potency, of cannabis, supplemented with sensitive assays of urine cannabinoids.^25^ Because there was no placebo cannabis and because all participants sought cannabis as a potential therapy for their symptoms, the trial design created bias toward a treatment effect that was attributable to expectancy, strengthening our confidence in the null findings for pain, depression, or anxiety symptoms. The inclusion criteria were based on self-reported symptoms and as such may not be generalizable to those with a formal diagnosis of primary insomnia, an anxiety disorder, or major depressive disorder. The study sample was relatively homogeneous in race and ethnicity and educational attainment. There was a high dropout rate in the immediate card acquisition group between randomization and baseline, reflecting the cost and administrative burden of obtaining a medical marijuana card. Although retention after randomization was higher in the delayed card acquisition group, retention from baseline was high in both groups.

## Conclusions

In this RCT, ownership of a medical marijuana card led to rapid onset and increased incidence and severity of CUD in some participants, particularly those with a chief concern of anxiety or depressive symptoms. The self-reported improvement in sleep quality warrants further study into the benefits of medical marijuana card ownership for insomnia and the risk of CUD. There were no observed benefits of obtaining a medical marijuana card for pain, anxiety, or depressive symptoms.

## Notes

Supplement 1.
Trial Protocol

Supplement 2.
eMethods.
**eTable 1.** Medical and Psychiatric History of the Overall Study Sample **eTable 2.** Concomitant Medications at Enrollment by Intervention Group **eTable 3.** Self-Reported CBD:THC Ratio and Method of Use by Intervention Group **eTable 4.** Cannabinoid Metabolite Measurements at Week 12 by Intervention Group **eTable 5.** Secondary Outcomes (SF-12 Mental and Physical Well-Being, and Cognitive Outcomes) by Intervention Group **eTable 6.** CUD Diagnosis by Group and Study Timepoint **eTable 7.** Cannabis Use Disorder Symptoms Endorsed in Each Group **eTable 8.** Exploratory Cannabis Use and Symptom Rating Outcomes **eTable 9.** Sample Characteristics at Screening Those Randomized to MMC Who Obtained vs Did Not Obtain MMC **eTable 10.** Sensitivity Analysis of Primary Outcomes by Randomization Group With Adjustment Using Propensity Scores **eTable 11.** Sensitivity Analysis of Primary Outcomes in MMC Participants With Metabolites, and in WLC Participants Without Detectable Metabolites **eTable 12.** Primary Outcomes in Participants With Blinded Assessments **eTable 13.** Treatment Emergent Adverse Events **eTable 14.** Severity of Treatment Emergent Adverse Events **eFigure.** CUD Symptoms by Intervention Group at Each Study Timepoint
eReferences

Supplement 3.
Data Sharing Statement

## References

[1] Whiting PF, Wolff RF, Deshpande S, . Cannabinoids for medical use: a systematic review and meta-analysis. JAMA. 2015;313(24):2456-2473. doi:10.1001/jama.2015.6358 26103030

[2] National Academies of Sciences, Engineering, and Medicine; Health and Medicine Division; Board on Population Health and Public Health Practice; Committee on the Health Effects of Marijuana: An Evidence Review and Research Agenda. *The Health Effects of Cannabis and Cannabinoids. The Current State of Evidence and Recommendations for Research*. National Academies Press; 2017. 28182367

[3] Reinarman C, Nunberg H, Lanthier F, Heddleston T. Who are medical marijuana patients? population characteristics from nine California assessment clinics. J Psychoactive Drugs. 2011;43(2):128-135. doi:10.1080/02791072.2011.587700 21858958

[4] Hasin DS, Saha TD, Kerridge BT, . Prevalence of marijuana use disorders in the United States between 2001-2002 and 2012-2013. JAMA Psychiatry. 2015;72(12):1235-1242. doi:10.1001/jamapsychiatry.2015.1858 26502112 PMC5037576

[5] González S, Cebeira M, Fernández-Ruiz J. Cannabinoid tolerance and dependence: a review of studies in laboratory animals. Pharmacol Biochem Behav. 2005;81(2):300-318. doi:10.1016/j.pbb.2005.01.028 15919107

[6] Lichtman AH, Martin BR. Cannabinoid tolerance and dependence. Handb Exp Pharmacol. 2005;(168):691-717. doi:10.1007/3-540-26573-2_24 16596793

[7] Solowij N, Battisti R. The chronic effects of cannabis on memory in humans: a review. Curr Drug Abuse Rev. 2008;1(1):81-98. doi:10.2174/1874473710801010081 19630708

[8] Solowij N, Stephens R, Roffman RA, Babor T. Does marijuana use cause long-term cognitive deficits? JAMA. 2002;287(20):2653-2654. 12020296

[9] Volkow ND, Swanson JM, Evins AE, . Effects of cannabis use on human behavior, including cognition, motivation, and psychosis: a review. JAMA Psychiatry. 2016;73(3):292-297. doi:10.1001/jamapsychiatry.2015.3278 26842658

[10] Haney M, Evins AE. Does cannabis cause, exacerbate or ameliorate psychiatric disorders? an oversimplified debate discussed. Neuropsychopharmacology. 2016;41(2):393-401. doi:10.1038/npp.2015.251 26286840 PMC5130141

[11] Schulz KF, Altman DG, Moher D; CONSORT Group. CONSORT 2010 statement: updated guidelines for reporting parallel group randomised trials. Int J Surg. 2011;9(8):672-677. doi:10.1016/j.ijsu.2011.09.004 22019563

[12] Sheehan DV, Lecrubier Y, Sheehan KH, . The Mini-International Neuropsychiatric Interview (M.I.N.I.): the development and validation of a structured diagnostic psychiatric interview for DSM-IV and ICD-10. J Clin Psychiatry. 1998;59(suppl 20):22-33. 9881538

[13] Ware J Jr, Kosinski M, Keller SDA. A 12-Item Short-Form Health Survey: construction of scales and preliminary tests of reliability and validity. Med Care. 1996;34(3):220-233. doi:10.1097/00005650-199603000-00003 8628042

[14] American Psychiatric Association. Diagnostic and Statistical Manual of Mental Disorders. 5th ed. American Psychiatric Association; 2013.

[15] Zigmond AS, Snaith RP. The hospital anxiety and depression scale. Acta Psychiatr Scand. 1983;67(6):361-370. doi:10.1111/j.1600-0447.1983.tb09716.x 6880820

[16] Cleeland CS, Ryan KM. Pain assessment: global use of the Brief Pain Inventory. Ann Acad Med Singap. 1994;23(2):129-138. 8080219

[17] Soldatos CR, Dikeos DG, Paparrigopoulos TJ. Athens Insomnia Scale: validation of an instrument based on ICD-10 criteria. J Psychosom Res. 2000;48(6):555-560. doi:10.1016/S0022-3999(00)00095-7 11033374

[18] Robbins TW, James M, Owen AM, Sahakian BJ, McInnes L, Rabbitt P. Cambridge Neuropsychological Test Automated Battery (CANTAB): a factor analytic study of a large sample of normal elderly volunteers. Dementia. 1994;5(5):266-281. 7951684 10.1159/000106735

[19] Adamson SJ, Kay-Lambkin FJ, Baker AL, . An improved brief measure of cannabis misuse: the Cannabis Use Disorders Identification Test-Revised (CUDIT-R). Drug Alcohol Depend. 2010;110(1-2):137-143. doi:10.1016/j.drugalcdep.2010.02.017 20347232

[20] Heishman SJ, Evans RJ, Singleton EG, Levin KH, Copersino ML, Gorelick DA. Reliability and validity of a short form of the Marijuana Craving Questionnaire. Drug Alcohol Depend. 2009;102(1-3):35-40. doi:10.1016/j.drugalcdep.2008.12.010 19217724 PMC2694410

[21] Sullivan MJL, Bishop SR, Pivik J. The Pain Catastrophizing Scale: development and validation. Psychol Assess. 1995;7(4):524-532. doi:10.1037/1040-3590.7.4.524

[22] Cohen S, Kamarck T, Mermelstein R. A global measure of perceived stress. J Health Soc Behav. 1983;24(4):385-396. doi:10.2307/2136404 6668417

[23] Trivedi MH, Wisniewski SR, Morris DW, . Concise Health Risk Tracking Scale: a brief self-report and clinician rating of suicidal risk. J Clin Psychiatry. 2011;72(6):757-764. doi:10.4088/JCP.11m06837 21733476

[24] Guy W. Clinical global impressions. In: ECDEU Assessment Manual for Psychopharmacology. National Institute of Mental Health; 1976:218-222.

[25] Klawitter J, Sempio C, Mörlein S, . An atmospheric pressure chemical ionization MS/MS assay using online extraction for the analysis of 11 cannabinoids and metabolites in human plasma and urine. Ther Drug Monit. 2017;39(5):556-564. doi:10.1097/FTD.0000000000000427 28640062 PMC5600652

[26] Gilman JM, Schmitt WA, Wheeler G, . Variation in cannabinoid metabolites present in the urine of adults using medical cannabis products in Massachusetts. JAMA Netw Open. 2021;4(4):e215490. doi:10.1001/jamanetworkopen.2021.5490 33844003 PMC8042519

[27] Enomoto K, Adachi T, Yamada K, . Reliability and validity of the Athens Insomnia Scale in chronic pain patients. J Pain Res. 2018;11:793-801. doi:10.2147/JPR.S154852 29713192 PMC5907892

[28] Krebs EE, Bair MJ, Damush TM, Tu W, Wu J, Kroenke K. Comparative responsiveness of pain outcome measures among primary care patients with musculoskeletal pain. Med Care. 2010;48(11):1007-1014. doi:10.1097/MLR.0b013e3181eaf835 20856144 PMC4876043

[29] Moorey S, Greer S, Watson M, . The factor structure and factor stability of the hospital anxiety and depression scale in patients with cancer. Br J Psychiatry. 1991;158:255-259. doi:10.1192/bjp.158.2.255 1812841

[30] Cook RJ, Farewell VT. Multiplicity considerations in the design and analysis of clinical trials. J R Stat Soc Ser A Stat Soc. 1996;159(1):93-110. doi:10.2307/2983471

[31] Benjamini Y, Hochberg Y. Controlling the false discovery rate: a practical and powerful approach to multiple testing. J R Stat Soc Ser B Stat Methodol. 1995;57(1):289-300. doi:10.1111/j.2517-6161.1995.tb02031.x

[32] Lev-Ran S, Roerecke M, Le Foll B, George TP, McKenzie K, Rehm J. The association between cannabis use and depression: a systematic review and meta-analysis of longitudinal studies. Psychol Med. 2014;44(4):797-810. doi:10.1017/S0033291713001438 23795762

[33] Pacek LR, Martins SS, Crum RM. The bidirectional relationships between alcohol, cannabis, co-occurring alcohol and cannabis use disorders with major depressive disorder: results from a national sample. J Affect Disord. 2013;148(2-3):188-195. doi:10.1016/j.jad.2012.11.059 23260381 PMC3608823

[34] Degenhardt L, Hall W, Lynskey M, Coffey C, Patton G. The association between cannabis use and depression: a review of the evidence. In: Castle D, Murray RM, D'Souza DC, eds. Marijuana and Madness. Cambridge University Press; 2011:114-128.

[35] Martins SS, Gorelick DA. Conditional substance abuse and dependence by diagnosis of mood or anxiety disorder or schizophrenia in the US population. Drug Alcohol Depend. 2011;119(1-2):28-36. doi:10.1016/j.drugalcdep.2011.05.010 21641123 PMC3179836

[36] Mechoulam R, Fride E, Hanus L, . Anandamide may mediate sleep induction. Nature. 1997;389(6646):25-26. doi:10.1038/37891 9288961

[37] Prospéro-García O, Amancio-Belmont O, Becerril Meléndez AL, Ruiz-Contreras AE, Méndez-Díaz M. Endocannabinoids and sleep. Neurosci Biobehav Rev. 2016;71:671-679. doi:10.1016/j.neubiorev.2016.10.005 27756691

[38] Vaughn LK, Denning G, Stuhr KL, de Wit H, Hill MN, Hillard CJ. Endocannabinoid signalling: has it got rhythm? Br J Pharmacol. 2010;160(3):530-543. doi:10.1111/j.1476-5381.2010.00790.x 20590563 PMC2931554

[39] Haroutounian S, Arendt-Nielsen L, Belton J, . International Association for the Study of Pain Presidential Task Force on Cannabis and Cannabinoid Analgesia: research agenda on the use of cannabinoids, cannabis, and cannabis-based medicines for pain management. Pain. 2021;162(suppl 1):S117-S124. 34138827 10.1097/j.pain.0000000000002266 PMC8855877

[40] Huang WJ, Chen WW, Zhang X. Endocannabinoid system: role in depression, reward and pain control (Review). Mol Med Rep. 2016;14(4):2899-2903. doi:10.3892/mmr.2016.5585 27484193 PMC5042796

[41] Woodhams SG, Sagar DR, Burston JJ, Chapman V. The role of the endocannabinoid system in pain. Handb Exp Pharmacol. 2015;227:119-143. doi:10.1007/978-3-662-46450-2_7 25846617

[42] Kim JH, Weinberger AH, Zhu J, Barrington-Trimis J, Wyka K, Goodwin RD. Impact of state-level cannabis legalization on poly use of alcohol and cannabis in the United States, 2004-2017. Drug Alcohol Depend. 2021;218:108364. doi:10.1016/j.drugalcdep.2020.108364 33143941

[43] Loflin MJE, Kiluk BD, Huestis MA, . The state of clinical outcome assessments for cannabis use disorder clinical trials: a review and research agenda. Drug Alcohol Depend. 2020;212:107993. doi:10.1016/j.drugalcdep.2020.107993 32360455 PMC7293929

