# Supplementary material for: Effect of Medical Marijuana Card Ownership on Pain, Insomnia, and Affective Disorder Symptoms in Adults: A Randomized Clinical Trial
Source: JAMA Netw Open. 2022 Mar 18;5(3):e222106. doi: 10.1001/jamanetworkopen.2022.2106 (PMC8933735; doi:10.1001/jamanetworkopen.2022.2106)
Supplement: Supplement 1. — Trial Protocol [file jamanetwopen-e222106-s001.pdf]

PARTNERS HUMAN RESEARCH COMMITTEE

DETAILED PROTOCOL: Effect of Medical Marijuana on Neurocognition and Escalation of Use
Protocol #: 2015P001600
Principal Investigator: Jodi Gilman, Ph.D.
Co-Investigator: A. Eden Evins, MD

Summary of Changes Made and Rationale

The IRB protocol, attached, was written for a pilot phase of the trial, and was updated when we received the R01. All measures, outcome definitions, and sample size calculations were up to date in clinicaltrials.gov (NCT03224468). The statistical analysis section was amended before any data were analyzed except for the propensity score analysis for missing data, explained below. No changes were changed after looking at the data, except for the propensity score analysis.

Method of randomization:
In Year 1 of the study, we realized that many participants, who were interested in obtaining an MMC never obtained one because of barriers such as expense or needing to go outside the usual healthcare system. We therefore changed our randomization from 1:1 to 2:1 MMC:WLC early in the trial in order to have enough participants in the MMC arm.

Outcomes and outcome definitions:
Outcome measures and definitions were updated on 07/19/2017 in clinicaltrials.gov (NCT03224468), but were not explicated stated in the IRB protocol.

Primary Outcome Measures:

- Cannabis Use Disorder Symptoms (*Time Frame: Baseline to 12 Weeks*)
  - The DSM-5 Cannabis Use Disorder Checklist will evaluate symptoms of CUD (number of symptoms).
- Changes in Symptoms of Depression and Anxiety (*Time Frame: Baseline to 12 Weeks)*
  - For those with depression and anxiety symptoms, the Hospital Anxiety and Depression Scale will be used to assess symptoms.
- Changes in Pain *(Time Frame: Baseline to 12 Weeks)*
  - For those with pain, the Brief Pain Inventory Severity Scale (BPI-S) will be used to assess levels of pain.
- Changes in Sleep *(Time Frame: Baseline to 12 Weeks)*
  - For those with insomnia, the Athens Insomnia Scale will be used to assess symptoms.

Secondary Outcome Measures

  - General Medical/Psychiatric Health (*Time Frame: Baseline to 12 Weeks*) The Short Form-12 Health Survey (SF-12) will be used to assess self-report of emotional and physical functioning.
- Cognitive Testing (*Time Frame: Baseline to 12 Weeks*)
  - Verbal learning/memory, attention, working memory, and complex decision-making will be assessed.
- Brain-based Measures (*Time Frame: Baseline to 1 Year*)

- Changes in resting state functional connectivity and task-associated activation in brain regions involved in reward, inhibitory control, and working memory will be assessed.

The IRB protocol includes exploratory outcomes that were not presented in this manuscript, but will be included in future publications.

Statistical Analysis: The IRB protocol did not include the final statistical plan. When we brought Dr. David Schoenfeld, an experienced clinical trial statistician, on this project in Year 2 of the study, well before any analysis had been conducted, he suggested that instead of a random slopes analysis that assumes the effect of the drug will be to gradually increase the effect or outcome, ie a time x treatment interaction, he suggested we should assume a constant treatment effect over the follow up period, 2 weeks, 1 month, 3 months. Dr. Schoenfeld also advised us not to adjust for covariates as we originally proposed to do, but to only adjust for baseline symptoms, since randomization should have accounted for differences and we had no strong prior information about what covariates would have an important effect on CUD in people seeking cannabis for medical complaints. Dr. Schoenfeld, without access to the data, made these recommendations to Drs Gilman and Evins after which Dr. Potter, who had access to the data, implemented the analyses.

Propensity score analysis for missing data: We could not ensure that participants in the MMC condition obtained an MMJ card, and we could not give participants a MMJ card because of state and hospitals regulations, a stated limitation of the study. Thus, we conducted a post hoc sensitivity analysis to verify the positive findings of the primary analysis in the final stages of the analysis when we realized that there was a problem with differential drop-out between the randomization and the baseline visit in the MMC and WLC groups.

Sample size calculation:
This was not included in the trial protocol but calculated for the R01 grant submission in 2016. We aimed to recruit 200 participants in order to have sufficient power to detect benefits and risks of MMCs. To identify a difference in development of CUD symptoms in the MMC vs WLC groups, assuming that the mean number of symptoms for MMC would be .4 (corresponding to 20% of subjects developing CUD) versus .1 in WLC (corresponding to 5% of subjects developing CUD), at a two-sided 0.05 significance level, power was 85%. To identify a difference in symptoms of pain, insomnia, depression, and anxiety, assuming a clinically significant effect would be a 30% reduction in presenting medical symptom with MMC while WLC subjects were expected to have a minimal change, assumed to be a 5% reduction, at a two-sided 0.05 significance level, based on effect sizes in the literature, power was 84% (pain), 90% (insomnia), and 84% (depression and anxiety), with sample sizes of approximately 1/3 (33 in each group) in the pain, insomnia, and anxiety/depression subgroups.

PARTNERS HUMAN RESEARCH COMMITTEE

DETAILED PROTOCOL: Effect of Medical Marijuana on Neurocognition and Escalation of Use
Protocol #: 2015P001600
Principal Investigator: Jodi Gilman, Ph.D.
Co-Investigator: A. Eden Evins, MD
Version Date: 04/15/2020

I. BACKGROUND AND SIGNIFICANCE

Cannabis is the most commonly used illicit substance in the United States, particularly among young people, and prevalence estimates are steadily increasing [1]. Recent estimates suggest that 52% of 18 to 25 year olds have ever tried cannabis[2], and 23.4% of high school students reporting use one or more times per month[3]. Delta-9-tetrahydrocannabinol (THC), the psychoactive compound in cannabis, binds to endogenous cannabinoid CB1 receptors located in brain regions such as the hippocampus, amygdala, basal ganglia, prefrontal cortex, substantia nigra and globus pallidus [4,5], making frontal-limbic neurocircuitry particularly susceptible to cannabis-related effects in the brain[6]. There is now a well-established relationship between regular cannabis use and a range of potentially adverse outcomes, including effects of learning and cognition (see [7,8] for review), effects on brain structure and function (see [9] for review), and potential impacts on mental health ([10]). Furthermore, regular cannabis use can lead to the development of cannabis abuse or dependence [[11,12]], particularly among daily users, where it is estimated that between 25-50% of users will develop a substance use disorder ([13]). A recent study found that younger age of onset of regular and maximum daily use were correlated with lower achievement scores and worse neurocognitive functioning[14].

At the time of this submission, 23 states have legalized marijuana for medical use. Virtually nothing is known about the effects of marijuana on those who choose to use marijuana for medical, and not recreational, purposes. Healthcare providers will be increasingly confronted with patients who are interested in using medical marijuana (MM) to treat various disorders, and few evidence-based studies exist to offer guidance regarding side effect profiles for these patients. Basic behavior regarding use of MM, including whether patients substitute MM for traditional medicines, resulting in reductions in use of opiates or other medications, is not known. Little is known about tolerability, amounts, duration of use, or whether patients reach a stable and regular dose of MM. As extensive research has shown that marijuana can affect memory, attention, decision-making, and even IQ, it is important to study whether or not those who use MM experience neurocognitive harms. Data is also lacking on whether those who use MM daily for chronic conditions also develop similar rates of addiction to those who use marijuana daily for recreational purposes.

It is important to study, in a rigorous and well-controlled manner, (1) basic behavior of MM patients, including general patterns of MM use, tolerability, dose (e.g. stable vs escalation of use), perception of benefit for presenting medical condition, and change in maintenance medication use (e.g. reduction/discontinuation in opiate use), (2) progression to addiction and escalation of use, including an experience loss of control over intake of marijuana or other substances, development of tolerance, marijuana caving, and experience of reinforcing effects (e.g. euphoria or 'high'), and withdrawal among those who choose to stop using marijuana, and (3) side effect profiles of medical marijuana on neurocognition, including memory, attention, and executive function, as well as possible effects on neural structure and function.

II. SPECIFIC AIMS

Aim 1: To observe behavior of MM patients in first months of use, including potential progression to addiction. We will examine, using rigorous real time daily dosing/purchase diaries, temporal patterns of MM use and change over time (e.g. stable vs escalation of use), tolerability, perception of benefit for presenting medical condition using EMA techniques, and change in

dose/frequency, type of maintenance medication use (e.g. reduction/discontinuation in opiate use) also with real time, daily diaries. We will also examine whether or not MM patients experience loss of control over intake of marijuana or other substances. We will use measures such as development of tolerance, escalation of use, marijuana caving, and assess experience of reinforcing effects (e.g. euphoria or 'high'), and withdrawal among those who choose to stop using marijuana.

Any changes throughout 12 months in perception of disease symptomatology or in use of maintenance medication will be compared to the waitlist control group.

Aim 2: To assess neurocognitive and psychiatric effects of MM. We expect that neurocognitive outcomes, such as working memory, attention, executive function, anhedonia, amotivation, and decision-making, (when not acutely intoxicated) may be worsened with MM use. Outcomes may be worse in those who use more frequently/heavily (as measured by quantitative THC screens and dosing dairies).

Aim 3: In a subset of patients, assess how MM effects neurocircuitry of prefrontal function. We will use functional MRI tasks to assess working memory, inhibitory control, sustained attention, and risk/reward task performance and brain activation patterns. We expect that individuals who use MM may experience changes from baseline in neurocircuitry involved in memory, attention, inhibitory control and reward.

Aim 4: To assess how medical marijuana patients compare to healthy controls in structural and functional brain imaging measures. We will use functional MRI tasks to assess memory, inhibitory control, sustained attention, and risk/reward task performance and brain activation patterns. We expect that individuals who use MM to show alterations in task-related brain activity.

III. PARTICIPANT SELECTION

<u>Medical Marijuana Participants</u>

Inclusion Criteria:

1. Men and women aged 18-65 years, inclusive;
2. Competent and willing to provide written informed consent;
3. Desire to use medical marijuana for self-reported pain, sleep, or affective (mood and/or anxiety including PTSD) symptoms.
4. Not in possession of a medical marijuana card, but expressing intent to get one.
5. Able to communicate in English language.

Exclusion Criteria:

1. Current daily marijuana use (prior to enrollment)
2. Current substance use disorders (e.g. cocaine, opiate, stimulant). Light to moderate alcohol use is permitted (defined as 16 or less on the AUDIT), and nicotine dependence is permitted because of the high co-use of nicotine and marijuana. Participants cannot meet current SCID criteria for a use disorder on any illicit substance.
3. Pregnant (verified by a urine test).
4. Diagnosis of acute coronary syndrome, coronary thrombosis, myocardial infarction, cardiomyopathies and arrhythmias such as atrial fibrillation, ventricular tachycardia or fibrillation, thrombophilia such as Factor V Leiden, Prothrombin 20210, antithrombin deficiency, Antiphospholipid syndrome.
5. History of deep vein thrombosis (DVT), pulmonary embolism, stroke.
6. In the opinion of the investigator, not able to safely participate in this study because of any medical or psychological issues (e.g. psychosis) that might compromise their safety.

Healthy Control Participants

Inclusion Criteria:

1. Men and women aged 18-65 years, inclusive
2. Competent and willing to provide written informed consent
3. Able to communicate in English language
4. No contraindications for fMRI (see MRI safety screening form).

Exclusion Criteria:

1. Current or past history of major medical illness by self-report
2. History of diabetes, cardiovascular disease, HIV, Hepatitis C, migraines, head injury or prolonged unconsciousness (> 24 hours)
3. Current use of drugs/medications, such as analgesics
4. Current use of illicit drugs, verified with urine drug screen
5. Pregnancy, verified with urine screen
6. Meets DSM-5 criteria for psychological disorders (current)
7. Reports using marijuana in the past 3 months
8. Reports using marijuana approximately > 50 times in their lifetime
9. In the opinion of the investigator, not able to safely participate in this study.

Healthy Control Participants and Medical Marijuana Participants to Undergo MRI Scanning

Additional Inclusion Criteria for MRI studies (applicable for only the subset who will undergo neuroimaging)

1. Randomized to active MM group or scanned as healthy control participants.

Additional Exclusion criteria for MRI studies (applicable for only the subset who will undergo neuroimaging)

1. Known claustrophobia
2. Presence of electrically, magnetically, or mechanically activated implants (such as cardiac pacemakers) or intracerebral vascular clips
3. History of working with metal (e.g. shavings or fragments could be lodged in scalp or eye)
4. Pregnancy (if the subject cannot rule out the possibility of pregnancy, a pregnancy test will be conducted prior to the study)
5. In the opinion of the investigator, not able to safely participate in this study.

IV. RECRUITMENT METHODS

Participants will be recruited by study staff at the MGH Center for Addiction Medicine through advertising by email, web and bulletin board announcements posted within the local site network community. We will place a Partners HRC approved research study advertisement on relevant websites and in relevant print publications, as well as on the MBTA and on radio advertisements. We will also recruit from local clinics.

In accordance with NIH guidelines, efforts will be made to attain a mix of study participants, in terms of gender and racial/ethnic representation that is reflective of the population of the greater metropolitan area where the recruitment is taking place. For example, for the metropolitan Boston area we will attempt to recruit; 63% white, 26% African American and 11% other races. We anticipate that half of our participants will be male and half female and that we will have representation from Asian American, African American and Hispanic minority groups in our final cohort that is representative of that reported in census data for Greater Boston.

V. STUDY PROCEDURES

Healthy Control Participants

Screening: Participants who have expressed interest in participating in the study will undergo a brief phone screen to assess eligibility. We plan to phone screen about 100 participants. If they are eligible according to the phone screen, they will be scheduled for an in-person study visit. At this visit, a consent form will be reviewed with the study doctor. If participants are determined eligible at the end of this visit, they will then be scheduled for the MRI scan.

Study Design:

Visit I: Healthy Control Screen

Visit I of this study will take place outside of the scanner only. Participants will be recruited and scheduled for a behavioral session. In this session, we will test participants on a computer in order to optimize the parameters of the task.

Visit II. Healthy Control Imaging Procedure

In Visit II, participants who have met all inclusion criteria and none of the exclusion criteria will be scheduled for an MRI scan. Each scan session will last up to two hours during which structural and/or functional images will be collected.

Participants will be positioned in the scanner and outfitted with audio and visual presentation and response indicator equipment. Visual images and auditory stimuli will be presented using MRI compatible presentation software and hardware. Participant responses are monitored using MRI-compatible keypads, and reaction times may be recorded for subsequent analysis of timing and accuracy of task performance.

Measure	Instrument	Visit I Screen	Visit II MRI Scan
**General Medical/Psychiatric Health**			
Medical History/ CON MEDS		x	
Drug Screen/pregnancy		x	x
Psychiatric Interview	MINI 7	x	
Suicidality and Health Risk Taking (N/A for scores < 9 on MINI Suicidality)	CHRT	x	
Provider Expectations for Recovery Scale	PERS	x	
**Substance Use**			
Frequency of substance use	TLFB (MJ, EtOH, tobacco, other drugs)	x	
Harmful drinking	AUDIT	x	
**Cognitive Testing**			
Intelligence / reading	WTAR	x	
**MRI Scan**			
MPRAGE	Structural		x
Resting state	Functional connectivity		x
Task-based (i.e. working memory, inhibitory control)	Task-based fMRI		x

MRI Protocol: The purpose of the MRI portion of the study is to compare the brains of individuals with no serious medical conditions, current psychological conditions, or drug use to those enrolled in the medical marijuana study. All of these participants will undergo a 3T MRI scanning. Each scan session will last up to two hours during which structural and functional data will be collected.

Participants will be positioned in the scanner and outfitted with audio and visual presentation and response indicator equipment. Visual images and auditory stimuli will be presented using MRI compatible presentation software and hardware. Participant responses are monitored using MRI-compatible keypads, and reaction times may be recorded for subsequent analysis of timing and accuracy of task performance.
Participants may undergo the scans noted below:

1. **Structural MRI**
For the structural imaging sessions T1 -weighted sequences may be acquired. Multiple anatomical imaging sequences may be run to assess specific image quality measures. The same anatomical scans will be repeated varying a limited range of pulse sequence parameters to assess their effect on the image contrast, image artifacts and signal to noise ratio. Parameters that will be varied may include the repetition time of the MR acquisition (TR), the echo time of the acquisition (TE), and the presence of pre-acquisition inversion pulses. The above parameters will also be evaluated in pulse sequences which acquire parameters used in the reconstruction of the MR images. The software and hardware interlocks put in place by the scanner manufacturer ensures that the range of these parameters will be maintained within the FDA's non-significant risk criteria.

2. **Functional Magnetic Resonance (fMRI)**
The scanner is outfitted with audio and visual presentation and response indicator equipment. All tasks are programmed using presentation software that is synchronized with the video synch pulse of the computer running the task. The presentation software also collects the behavioral response information. The experimental paradigms will assess activation in brain regions underlying memory, attention, inhibitory control, and risk/reward. Participants may earn points for correct answers, and those points may be converted to monetary values. The experimental paradigm will take approximately 45 minutes to complete. We will also assess resting-state functional connectivity using standard FC-MRI sequences. The entire scan will last about one hour.

Drug Testing: At both study visits, participants will provide a urine sample, which will be used to qualitatively screen for cannabis use as well as other substances of abuse. An alcohol reading may also be taken if there is any concern about a participant being intoxicated.

<u>Medical Marijuana Participants</u>
Screening: Participants who have expressed interest in participating in the study will undergo a brief phone screen to assess eligibility. We plan to phone screen about 2000 participants. If they are eligible according to the phone screen, they will be scheduled for an in-person (or teleconference) study visit. At this visit, a consent form will be reviewed with the study staff. Participants who give permission to receive text messages from the study staff will receive appointment reminders via text two days before upcoming appointments. In addition, subjects will be instructed on how to use our phone app for tracking their medical marijuana use, they will fill out some questionnaires and also do a urine drug and if applicable, a pregnancy test. Also at this visit, participants will be randomized to either the active study group or the waitlist control group. We plan to randomize 200 participants to each group.

Study Design: The active study group will be asked to call the study staff once they receive their MM cards. A staff member will follow up via phone with participants who do not contact study staff within approximately two weeks after the screening visit. Staff will contact participants again after approximately four weeks and six weeks to check in on the status of the medical marijuana card application. If after six weeks, a participant still has not made any progress on the application for a

medical marijuana card (i.e., made an appointment with a doctor who recommends medical marijuana), the participant will be discontinued from the study and a three-month follow-up visit will be scheduled.

When participants in the active study group receive their medical marijuana card, they will be scheduled for a baseline assessment in which they will undergo cognitive testing, interviews, questionnaires, and possibly an MRI scan (see Measures below). In contrast, participants in the waitlist control group will be scheduled for the baseline visit approximately 3-4 weeks following the screening visit. All participants will then be scheduled for repeat assessments at approximately 2 weeks, 1 month, 3 months, 6 months and 1 year after the baseline visit. All participants will receive monthly check in phone calls from a research coordinator at approximately 2, 4, 5, 7-11 months past baseline in order to update study staff on medication changes, recent substance use, and overall physical and mental health. Participants will also be asked to complete a 2-year follow-up phone call, which is identical to previous check in calls, with the addition of the CGI and CUD checklist. If participants are in the active study group, we will ask that they not use MM on the day of testing (though they can use it after testing on that day). These visits will take approximately 3 hours to complete.

In consultation with their prescribing physician, participants in the waitlist control group will be asked to wait to start using MM until they have completed Visit 5 (Three Months). During those 3 months, they will be assessed at the same time points as those in the active MM group (see study schema). After the 3-month waitlist period, the waitlist control group will have the option to start using MM. If this group fails to adhere to the waitlist control, they will be kept in the study and will be asked to complete the same assessment schedule as those initially assigned to the active study group. All participants will be assessed after 1 year.

monthly check in phone calls ↓ ↓ ↓ 2-yr follow-up call ↓

Measure	Instrument	Visit 1 Screen	Visit 2 Baseline	Visit 3 Two Weeks	Visit 4 One Month	Visit 5 Three Months	Visit 6 Six Months	Visit 7 One Year
**General Medical/Psychiatric Health**								
Medical History/ CON MEDS		x	x	x	x	x	x	x
Quantitative cannabinoids			x	x	x	x	x	x
Drug Screen/pregnancy		x	x	x	x	x	x	x
Psychiatric Interview	MINI 7 + Past SUD and AUD	x						x
Suicidality and Health Risk Taking	CHRT	x	x	x	x	x	x	x
Emotional and physical Functioning	SF-12		x	x	x	x	x	x
ADHD symptoms	ASRS		x					x
Impulsivity	BIS-11		x			x	x	x
Executive function	BRIEF		x			x	x	x
Risk perceptions and behavior	DOSPERT		x			x		
Family history of Addiction	KSADS	x						
Provider Expectations for Recovery Scale	PERS	x						
**Substance Use**								
Frequency of substance use	TLFB (MJ, EtOH, tobacco, other drugs)	x	x	x	x	x	x	x

Cannabis Use Disorder Screen	DSM-5 Cannabis Use Disorder Checklist	x	x	x	x	x	x	x
MJ craving	MCQ-SF		x	x	x	x	x	x
Marijuana Motives	MMM					x		
Marijuana Withdrawal (if stop)	CWS		x	x		x	x	x
MJ use severity	CUDIT		x	x	x	x	x	x
Harmful drinking	AUDIT	x				x	x	x
Alcohol consumption urge	URGE		x	x	x	x	x	x
Expectancy of MMJ effects	MMEEQ	x	x		x	x	x	x
Reflection of MMJ effects (qualitative)							x	x
**Symptoms**								
Pain level	BPI	x	x	x	x	x	x	x
Pain Catastrophizing Scale	PCS	x	x	x	x	x	x	x
Perceived Stress Scale	PSS	x	x	x	x	x	x	x
Clinician's Severity Scale	CGI		x	x	x	x	x	x
Insomnia	AIS	x	x	x	x	x	x	x
Anxiety Scale	HADS	x	x	x	x	x	x	x
Delusions and Psychosis	PDI		x		x	x	x	x
Difficulties due to health conditions	WHODAS	x	x			x	x	x
Post-traumatic stress symptoms	PCL	x						x
**COVID-19 Specific Questionnaires**								
Remote Pre-Visit Form	PVF	x	x	x	x	x	x	x
Effects of COVID-19 pandemic on mood, pain, sleep, lifestyle	COVID Impact	x	x	x	x	x	x	x
Impact of epidemic/pandemic on various areas of life	EPII	x						
Changes in substance use since COVID-19 pandemic	Covid19-CSU	x						
**Cognitive Testing**								
Verbal learning/memory, attention, working memory, and complex decision-making	CANTAB		x		x	x	x	x
Intelligence / reading	WTAR	x						
**MRI Scan**								
MPRAGE	Structural		x					x
Resting state	Functional connectivity		x					x
Task-based (i.e. working memory, inhibitory control)	Task-based fMRI		x					x

Drug Testing: At all study visits, participants will provide a urine sample, which will be used to qualitatively screen for cannabis use as well as other substances of abuse. For participants who provide written consent for sample shipment, urine samples will be shipped to quantitatively screen for amount of THC metabolites in urine. Samples will only be shipped for participants who report recent marijuana use and/or qualitatively screen positive for THC. An alcohol reading may also be taken if there is any concern about a participant being intoxicated.

Urine Collection During Remote Data Collection: While we must conduct remote study visits, participants will be asked to consent to shipping their urine samples to study staff for analysis of cannabinoid metabolites. Participants who do not consent to remote urine collection will not be penalized and will not receive materials for shipping their urine for testing. Participants who consent

will receive urine sample kits by mail prior to their remote visits. Kits include a sample cup, biohazard bag, packaging bag, UPS shipping bag, and detailed instructions on how to provide and package their urine sample. Participants will be instructed to leave the packaged sample at the location where their mail is picked up at their home before the scheduled pickup time. Because there is no staff at our MGH offices, study staff will schedule a UPS pickup for overnight shipping from the participant's home to the home of our Center Director, Dr. Eden Evins, MD, who will be available to transport urine samples to the -80 degree freezer in the Thier Building on the MGH campus for storage prior to batch transport to Colorado for analysis of cannabinoid metabolites central to understanding the primary outcomes of the study. Dr. Evins will pipette aliquots of samples into labeled vials for freezer storage. Samples will be batch shipped on dry ice to Colorado when that lab is again functioning at its capacity and able to conduct quantitative assay for cannabinoid metabolites per study protocol. Dr. Evins is approved as Emergency Level 4 Research Staff Essential Personnel, and as such has access to the -80 degree freezer in the Their Research building, as per guidance from hospital leadership. Drs. Gilman and Evins, and 2 other members of study staff also have this approval and can take over this urine aliquoting and transport task should Dr. Evins be unable to perform it due to illness or redeployment.

Online Dosing Diaries: All participants will be asked to keep a daily log of (1) marijuana use, and (2) ratings of pain, sleep quality, mood, and general health. They will be asked to send in data once per day during the first three months of study participation (from Visit 1 to Visit 5). We will collect this data using Secure Survey, a web application designed to collect data from a modern user group on their mobile, tablet, or desktop devices.

MRI Protocol (Optional): The purpose of the MRI portion of the study is to investigate possible changes in the brain from those who obtain MM cards. Therefore, only patients randomized the active MM group will be given the option to undergo a 3T MRI scanning. Participants will undergo MRI scanning at baseline and at 1 year after initiation of MM. In addition, a small group (n = 25) of non-MJ-using controls, matched on demographics, will also be scanned at baseline and after 1 year in order to allow us to assess normative change over time. Each scan session will last up to two hours during which structural and functional data will be collected. Participants will be positioned in the scanner and outfitted with audio and visual presentation and response indicator equipment. Visual images and auditory stimuli will be presented using MRI compatible presentation software and hardware. Participant responses are monitored using MRI-compatible keypads, and reaction times may be recorded for subsequent analysis of timing and accuracy of task performance.
Participants may undergo the scans noted below:

1. **Structural MRI**
   For the structural imaging sessions T1 -weighted sequences may be acquired. Multiple anatomical imaging sequences may be run to assess specific image quality measures. The same anatomical scans will be repeated varying a limited range of pulse sequence parameters to assess their effect on the image contrast, image artifacts and signal to noise ratio. Parameters that will be varied may include the repetition time of the MR acquisition (TR), the echo time of the acquisition (TE), and the presence of pre-acquisition inversion pulses. The above parameters will also be evaluated in pulse sequences, which acquire parameters used in the reconstruction of the MR images. The software and hardware interlocks put in place by the scanner manufacturer ensure that the range of these parameters will be maintained within the FDA's non-significant risk criteria.

2. **Functional Magnetic Resonance (fMRI)**
   The scanner is outfitted with audio and visual presentation and response indicator equipment. All tasks are programmed using presentation software that is synchronized with the video

synch pulse of the computer running the task. The presentation software also collects the behavioral response information. The experimental paradigms will assess activation in brain regions underlying memory, attention, inhibitory control, and risk/reward. Participants may earn points for correct answers, and those points may be converted to monetary values. The experimental paradigm will take approximately 45 minutes to complete. We will also assess resting-state functional connectivity using standard FC-MRI sequences. The entire scan will last about one hour.

V. REMUNERATION

Healthy Control Participants

Participants in the healthy control group will be paid for the completion of each study visit. Each study visit is compensated in the amount of $50. If participants undergo the screening visit but are not eligible, they will be paid $20 for the first visit only. There are opportunities to receive additional money in the MRI scanner ($1- $50) based on scan task performance. All participants who drive to visits will also be given parking vouchers for each visit. This level of compensation is deemed appropriate given the time commitment. All payments will be made via check following each study visit.

Medical Marijuana Participants

Participants in the medical marijuana study will be paid after the completion of each study visit, and weekly for submitting their dosing diaries. Participants will be compensated $20 on the day of the screen and $60 for each study visit. Participants will be paid an additional $40 after completing their six month visit and an additional $60 after completing their twelve month visit. Participants will be paid $10 for completing seven monthly 10-minute check in phone calls at months two, four, seven, eight, nine, ten and eleven with a research coordinator. The purpose of these phone calls is to record AEs, medication changes, TLFB, and symptom severity (measured by the SF-12). Participants will be paid an additional $5 for completing the EPII and $5 for completing the Covid19-CSU. If participants undergo the screening visit but are not eligible, they will be paid $20 for the first visit only. They will be paid $20 per week for submitting dosing diaries during the first three months of the study, for a maximum total of $260 for completing all dosing diaries. Additionally, they may be paid for pre-baseline daily diary entries for a maximum of five weeks ($20 per week, $100 total). They will be paid $50 for each scan if they agree to undergo scanning procedures. For each scan participants may also receive additional money ($1-100) based on scan task performance. All participants who drive to visits will also be given parking vouchers for each visit. All payments will be made via check at the end of the study.

Please see table below for payment schedule.

Visit #	Active Study Group		Waitlist Control Group	
1	Screening	$20	Screening	$20
2	Baseline	$60	Baseline	$60
3	2 weeks	$60	2 weeks	$60
4	1 month	$60	1 month	$60
5	3 months	$60	3 months	$60
	Dosing Diaries (13 weeks x $20/week)	$260	Dosing Diaries (13 weeks x $20/week)	$260
6	6 months	$100	6 months	$100

	Monthly phone calls 2, 4, 7-11 months (7 x $10 each)	$70	Monthly phone calls 2, 4, 7-11 months (7 x $10 each)	$70
	Covid19 Questionnaires	$10	Covid19 Questionnaires	$10
**12**	12 months	$120	12 months	$120
**Total**		$820	Total	$820
Scan (Optional)	Baseline	$50		
	12 months	$50		
**Total**		$920		

VI. PARTICIPANT ENROLLMENT

MGH Study staff will conduct telephone screening of potential subjects. A telephone screening will distinguish the majority of potentially eligible subjects from those not meeting eligibility criteria. This will consist of a brief discussion of the research study, confirming a potential participant's understanding of the basic study procedures, interest in participation and whether he/she meets eligibility criteria and includes asking for current medications, gender, age, history of claustrophobia, pregnancy status, history of psychiatric conditions including substance use disorders. Screening takes place over the phone in response to a potential participant inquiry (**see attached screening telephone script**). Those not eligible for the experiment based on the phone screen will be informed that they do not qualify for entry into this particular study. An initial cohort of up to 2000 adults will be recruited, and 400 will be enrolled. The research study consent form will be made available to the participants on their study visit before any study procedures occur.

A non-identifying prescreening log will be kept for all individuals screened. The screening log will include a non-identifying subject ID, the date of screening, whether eligibility criteria were met, reason for exclusion, and enrollment decision or status.

VII. INFORMED CONSENT

Participants who are deemed eligible on the basis of the phone screen will be asked to come in or to participate in a teleconference for a study visit. At the time of such visit, informed consent will be obtained by a trained member of the study staff with investigator back-up, prior to administering any study procedures. All participants will be given the opportunity to ask questions to a doctoral-level member of study staff or an Investigator during the consent process. Contact information of key study staff will be provided and participants will be informed that the investigators are available to answer any questions or concerns they may have about the study. All enrolled participants will be provided with a copy of their signed consent form.

VIII. PARTICIPANT WITHDRAWAL

Participation is voluntary, and participants may stop being in the study at any time or decide not to join the study. If a participant decides not to participate, they will not be penalized in any way, and will not lose any benefits to which they are otherwise entitled.

IX. CONFIDENTIALITY

All phases and aspects of this project will be conducted according to the Declaration of Helsinki, and will comply with HIPAA regulations. The sponsors of this research will only have access to data on individuals that is stripped of all unique identifiers according to HIPAA guidelines.

Biostatistical Analysis

Data variables collected for study

Standardized questionnaires, assessing drug use, personality characteristics, and anxiety will be collected. Cognitive data will be collected using standardized computer programs such as the CANTAB. MRI data will also be collected.

Data will be collected and managed using REDCap (Research Electronic Data Capture) tools (Harris 2009) hosted with Partners HealthCare. REDCap data collection projects rely on a thorough study-specific data dictionary defined in an iterative self-documenting process by all members of the research team with planning assistance from Partners HealthCare Research Computing, Enterprise Research Infrastructure & Services (ERIS) group. The REDCap Survey is a powerful tool for building and managing online surveys. The research team can create and design surveys in a web browser and engage potential respondents using a variety of notification methods. Participants will complete a number of REDCap Surveys using a tablet provided to them at study visits. This platform allows access to specific surveys and participants are required to initial at the completion of each. Study staff members will initial to indicate who completed each item. Both REDCap and REDCap Survey systems provide secure, HIPAA compliant, web-based applications that are flexible enough to be used for a variety of types of research, provide an intuitive interface for users to enter data and have real time validation rules (with automated data type and range checks) at the time of entry. Online dosing diaries will be collected using Secure Survey, a web application designed to collect data from a modern user group on their mobile, tablet, or desktop devices. This platform secures not only the data transmission, but also the storage using industry standard security techniques.

Analytic Plan

Power/Sample Size. Aim 1 seeks to address the development of CUD (dependent on the number of symptoms reported) in the MM compared with the WLC group. To assess this, we will use the DSM-5 criteria for CUD, which combines the DSM-IV categories of abuse and dependence into a single disorder measured on a continuum from mild to severe. We have based power sample size requirements on the between and within subject ANOVA of the dependent variable of CUD symptoms, comparing the MM versus WLC conditions tested across the baseline to 3 month periods, where the primary effect of interest is the condition X time interaction. A power analysis using the Gpower computer program[29] indicated that a sample of 80 participants per group would be needed to detect small to medium effect (Cohen's d=.3) with 90% power using a t test with alpha at .05.

Data Analysis Plan: In general, 3 analytic approaches will be employed, corresponding to three types of comparisons and data:

(a) Numeric Data Assessed at Baseline, 3 Months, ***6 Months and 1 year.*** Measures of addiction, tolerance, and cognitive performance will be analyzed with a between- and within-subject analysis of covariance, the primary between-subject factor being Condition, i.e., the active MM vs. WLC group, and the within-subject factor being time, i.e., assessments at baseline and 3 months. Relevant subject-level numeric covariates (i.e. THC metabolite levels) and categorical factors (e.g. disease category) crossed with condition and possibly interacting with time will be included in the analysis. Covariates such as baseline MJ use, primary disorder for which MM is sought, age, and gender will be of interest. The term of primary interest will be the interaction of condition x time as a test of the hypothesis that assignment to the MM condition will be associated with an increased rate of CUD. A limited amount of backward elimination of nonsignificant ancillary terms will be employed to increase power for effects of more interest (e.g. non-significant terms can be dropped from the models). An additional, analogous repeated-measure ANCOVA, restricted to the MM group, will include the third

assessment at 6 months. The Greenhouse-Geisser adjustment for correlated error will be employed for significance tests with more than one degree of freedom. For all models, residuals will be checked graphically for adherence to assumptions of normality and homoscedasticity, and data transformations applied if needed.

(b) Daily Numeric Data Collected via Dosing Diaries in the First 3 Months. Daily-recorded numeric measures such as dose and frequency of use of MJ, of analgesic, anxiolytic, and/or antidepressant medications, and ratings of pain, sleep, mood, and anxiety will be analyzed as dependent variables in separate analyses. Preliminary exploratory graphical analyses will be conducted using longitudinal "spaghetti plots" in order to check any need for curvilinear models, explore any clusters of trajectories, and address distribution problems such as floors/ceilings, non-normality, outliers, etc. Analysis methods employed will be mixed fixed and random effects longitudinal models. Fixed terms will be Condition (MM vs. WLC groups) and various relevant crossed factors and numeric covariates. Correlated random terms will be subjects' intercepts and linear rate of change of their trajectories on the dependent variable across days (curvilinear terms will be included if warranted). In order to test the predictive relation of some of the time-varying variables (e.g., frequency of MJ use) on others (e.g., pain recordings or use of analgesic, anxiolytic, and/or antidepressant medications), the former will be included as additional time-varying predictors in models otherwise analogous to those above. Exploratory structural equation models (SEM) and cross correlations of time series will be examined for evidence of underlying causal dynamics. Cross-lagged partial correlations can be used to test direction of effects.

(c) Imaging Data. Imaging data will be analyzed using standard analysis platforms for longitudinal analysis (e.g. FSL and SPM). We will use a linear mixed-effects model approach, with a univariate two-stage specification. The advantage of this method is that it offers flexibility in handling unbalanced repeated measurements with missing data, which is one of the biggest challenges to longitudinal fMRI datasets. At the subject-level, a unique trajectory for each individual is defined for each region or voxel being considered, meaning that activation measurements are condensed into summary statistics for each scan. At the second, population-level, individuals are considered as arising from a population of all such individuals, each with a unique intercept and slope, and a linear mixed-effects model is fit. With this modeling approach, it is reasonable to deduce that the within-individual variances are the fluctuations around the individual-specific trajectory, and the among-individual variances can be described as differences of parameters characterizing these trajectories. As one way to conduct this model using brain-imaging data, statistical measures of brain activity responses serve as the dependent variable and one linear mixed-effect model is then fit for each ROI or voxel. Repeatability of fMRI can be directly addressed via certain parameters of this model.

Risks and Discomforts

Psychosocial risks

Risks: There are no legal risks to the participants associated this study, because medical marijuana is legal in Massachusetts. There is a small social risk associated with participation in this study, as some people may be embarrassed if others found out that they are using medical marijuana. Minimization: All data will be completely deidentified and anonymized. Please see 'confidentiality and loss of privacy' below.

The Co-Investigators on this project include a psychiatrist (Dr. Eden Evins) and a clinically-trained clinical psychologist (Dr. Randi Schuster). If there are any concerns about a subject who is actively suicidal and in need of clinical attention, the PI will be made aware of the issue immediately and will consult with appropriate medical personnel (co-investigators Eden Evins and Randi Schuster) to determine appropriate steps. The PI and medically trained co-investigators will assess the needs of the subject and offer the subject either prompt treatment or medical referral, whichever is appropriate

for the situation. There is a licensed clinician on site 40 hours per week, and other MGH resources can be used as necessary.

Confidentiality and Loss of Privacy
Risks: The risk of loss of privacy is judged to be minimal. Online dosing diaries will be collected using Secure Survey, which secures not only the data transmission, but also the storage using industry standard security techniques.
Minimization: Confidentiality will be maintained by numerically coding all data and by keeping all data in password protected databases. Subject information will be accessible only to research staff. Information about study participants will not leave our institution in any form that would identify individual subjects.
Secure Survey implements multiple layers of security using industry standard techniques:

- SSL Certificates from Trusted Certificate Authorities encrypts all traffic between the user's device and the server
- All database data is encrypted using 256-bit encryption ensuring no data is at risk should the servers be breached
- Browser tokens further secure the flow between pages and help prevent XSS attacks

fMRI Risks
Risks: fMRI is a minimal risk procedure. Although fMRI scanning itself is painless, subjects may experience discomfort. Some may become claustrophobic inside the magnet. Subjects may also be bothered by the beeping and hammering sounds made when the scanner is collecting measurements, and/or experience peripheral stimulation, manifested as a gentle tap or sensation of mild electric shock. Because of the high magnetic field of the fMRI scanner, individuals with pacemakers, cosmetics, or certain metallic implants in their bodies must be excluded. Each potential subject must identify these and other possible contraindications prior to fMRI scanning. Because the scanner attracts certain metals, precautions must be taken to remove metallic objects from the MRI room.

Minimization: Since some subjects may be uncomfortable in the MRI, all subjects will be able to converse with the experimenter via a microphone and speaker system, and will be able to communicate an immediate need to come out or stop the scan via a "panic squeezeball". All efforts will be made to make the subjects as comfortable as possible while in the scanner. A member of the study staff will explain the procedure thoroughly to the subjects prior to scanning, allowing for maximal understanding and comfort. The MRI can be stopped at any time at the subjects' request. In addition, the scanners are equipped with an emergency button that the subject can press if necessary. A qualified M.D. will always be on site or reachable by pager in the unlikely event that an adverse event occurs. If there are any concerns about a subject in need of clinical attention, the PI will be made aware of the issue immediately and will consult with appropriate medical personnel (co-investigator: A. Eden Evins, MD) to determine appropriate steps.

Potential subjects will be carefully screened before recruitment with respect to their medical history. This includes: history of head trauma, surgical aneurysm clips, cardiac pacemaker or any other type of implants, metal rods or plates. They will be queried about their previous surgeries, hearing aid, intrauterine devices, dentures and pregnancy. Individuals with any one of these contraindications will not be recruited for the study.

If there are any concerns about a subject in need of clinical attention, the PI will be made aware of the issue immediately and will consult with appropriate medical personnel (co-investigator Eden Evins, MD) to determine appropriate steps. The PI and medically trained co-investigators will assess the needs of the subject and offer the subject either prompt treatment or medical referral, whichever is

appropriate for the situation. There is a licensed clinician on site 40 hours per week, and other MGH resources can be used as necessary.

Potential Benefits

Participants may find that talking about marijuana use increases their awareness of any issues related to drug use. Any participant who asks about treatment will be provided information regarding local drug treatment programs.

Information developed from this study may help researchers in the future. Specifically it is expected that this study will provide valuable information about effects and consequences of MM use, where few studies have been published. The proposed project will fill a critical gap in the addiction literature, at a critical time when cannabis is being legalized for 'medical' use with little known about effects of MM on target symptoms, addictions, neurocognition or brain structure and function.

MONITORING AND QUALITY ASSURANCE

All participants will have contact information for the principal investigator and the study coordinator if they have questions at any time. Dr. Gilman is responsible for the overall management of the study and will maintain regular communication with all of the study staff. The PI will meet weekly with all study investigators to review the details of data acquisition and analysis as well as any minor problems. In the event of any significant adverse event, the PI will be contacted immediately. The PI is responsible for the generation of summary reports to NIH documenting this process and outcome, which will also be included in the Continuing Review reports to the IRB.

Serious adverse events are not expected as a result of the study procedures. Should one occur, it will be reported by telephone or email by the principal investigator to the Partners IRB according to current PHRC Adverse Event Reporting Policy (version dated: March 13, 2007). All adverse events (if not serious) will be reported in writing to the Partner's Human Research Committee. All information regarding experimental subjects will be kept in the offices of the Principal Investigator. All data will be identified by a unique code number.

References

1. Substance Abuse and Mental Health Services Administration, Results from the 2013 National Survey on Drug Use and Health: Summary of National Findings, NSDUH Series H-48, HHS Publication No. (SMA) 14-4863. Rockville, MD: Substance Abuse and Mental Health Services Administration; 2014:31.
2. Substance Abuse and Mental Health Services Administration, Results from the 2013 National Survey on Drug Use and Health: Summary of National Findings, NSDUH Series H-48, HHS Publication No. (SMA) 14-4863. Rockville, MD: Substance Abuse and Mental Health Services Administration; 2014:25.
3. Kann LK, S.; Shanklin, S. L.; Flint, K.H.; Hawkins, J.; Harris, W.A.; Lowry, R.; O'Malley Olsen, E.; McManus, T.; Chyen, D.; Whittle, L.; Taylor, E.; Demissie, Z.; Brener, N.; Thornton, J.; Moore, J.; Zaza, S. Youth Risk Behavior Surveillance - United States, 2013. Rockville, MD: ICF International; Westat; 2014:20.

4. Mackie K. Distribution of cannabinoid receptors in the central and peripheral nervous system. *Handbook of experimental pharmacology.* 2005(168):299-325.
5. Piomelli D. The molecular logic of endocannabinoid signalling. *Nature reviews. Neuroscience.* 2003;4(11):873-884.
6. Martin-Santos R, Fagundo AB, Crippa JA, et al. Neuroimaging in cannabis use: a systematic review of the literature. *Psychological medicine.* 2010;40(3):383-398.
7. Solowij N, Battisti R. The chronic effects of cannabis on memory in humans: a review. *Current drug abuse reviews.* 2008;1(1):81-98.
8. Solowij N, Stephens R, Roffman RA, Babor T. Does marijuana use cause long-term cognitive deficits? *Jama.* 2002;287(20):2653-2654; author reply 2654.
9. Lorenzetti V, Solowij N, Fornito A, Lubman DI, Yucel M. The association between regular cannabis exposure and alterations of human brain morphology: an updated review of the literature. *Curr Pharm Des.* 2014;20(13):2138-2167.
10. Hall W, Degenhardt L. Adverse health effects of non-medical cannabis use. *Lancet.* 2009;374(9698):1383-1391.
11. Anthony JC. *Cannabis dependence: Its Nature, Consequences and Treatment.* Cambridge, UK: Cambridge University Press; 2006.
12. Lopez-Quintero C, Perez de los Cobos J, Hasin DS, et al. Probability and predictors of transition from first use to dependence on nicotine, alcohol, cannabis, and cocaine: results of the National Epidemiologic Survey on Alcohol and Related Conditions (NESARC). *Drug and alcohol dependence.* 2011;115(1-2):120-130.
13. DrugFacts: Marijuana. 2010; http://www.drugabuse.gov/publications/drugfacts/marijuana.
14. Hooper SR, Woolley D, De Bellis MD. Intellectual, neurocognitive, and academic achievement in abstinent adolescents with cannabis use disorder. *Psychopharmacology.* 2014;231(8):1467-1477.
15. Ware J, Jr., Kosinski M, Keller SD. A 12-Item Short-Form Health Survey: construction of scales and preliminary tests of reliability and validity. *Med Care.* 1996;34(3):220-233.
16. Kessler RC, Adler L, Ames M, et al. The World Health Organization Adult ADHD Self-Report Scale (ASRS): a short screening scale for use in the general population. *Psychological medicine.* 2005;35(2):245-256.
17. Patton JH, Stanford MS, Barratt ES. Factor structure of the Barratt impulsiveness scale. *Journal of clinical psychology.* 1995;51(6):768-774.
18. Weber EU, Blais, A., & Betz N.E. A Domain-specific Risk-attitude Scale: Measuring Risk Perceptions and Risk Behaviors. *Journal of Behavioral Decision Making.* 2002;15:263–290.
19. The Alcohol, Smoking and Substance Involvement Screening Test (ASSIST): development, reliability and feasibility. *Addiction.* 2002;97(9):1183-1194.
20. Sobell MB, Sobell LC, Klajner F, Pavan D, Basian E. The reliability of a timeline method for assessing normal drinker college students' recent drinking history: utility for alcohol research. *Addictive behaviors.* 1986;11(2):149-161.
21. Bohn MJ, Krahn DD, Staehler BA. Development and initial validation of a measure of drinking urges in abstinent alcoholics. *Alcoholism, clinical and experimental research.* 1995;19(3):600-606.
22. Adamson SJ, Sellman JD. A prototype screening instrument for cannabis use disorder: the Cannabis Use Disorders Identification Test (CUDIT) in an alcohol-dependent clinical sample. *Drug and alcohol review.* 2003;22(3):309-315.

23. Saunders JB, Aasland OG, Babor TF, de la Fuente JR, Grant M. Development of the Alcohol Use Disorders Identification Test (AUDIT): WHO Collaborative Project on Early Detection of Persons with Harmful Alcohol Consumption--II. *Addiction.* 1993;88(6):791-804.
24. Schafer J, Brown SA. Marijuana and cocaine effect expectancies and drug use patterns. *Journal of consulting and clinical psychology.* 1991;59(4):558-565.
25. Farrar JT, Young JP, Jr., LaMoreaux L, Werth JL, Poole RM. Clinical importance of changes in chronic pain intensity measured on an 11-point numerical pain rating scale. *Pain.* 2001;94(2):149-158.
26. Soldatos CR, Dikeos DG, Paparrigopoulos TJ. Athens Insomnia Scale: validation of an instrument based on ICD-10 criteria. *J Psychosom Res.* 2000;48(6):555-560.
27. Watson D, Weber K, Assenheimer JS, Clark LA, Strauss ME, McCormick RA. Testing a tripartite model: I. Evaluating the convergent and discriminant validity of anxiety and depression symptom scales. *Journal of abnormal psychology.* 1995;104(1):3-14.
28. Treadway MT, Buckholtz JW, Schwartzman AN, Lambert WE, Zald DH. Worth the 'EEfRT'? The effort expenditure for rewards task as an objective measure of motivation and anhedonia. *PloS one.* 2009;4(8):e6598.
29. Faul F, Erdfelder E, Lang AG, Buchner A. G*Power 3: a flexible statistical power analysis program for the social, behavioral, and biomedical sciences. *Behavior research methods.* 2007;39(2):175-191.
