# Supplementary material for: Effect of Medical Marijuana Card Ownership on Pain, Insomnia, and Affective Disorder Symptoms in Adults: A Randomized Clinical Trial
Source: JAMA Netw Open. 2022 Mar 18;5(3):e222106. doi: 10.1001/jamanetworkopen.2022.2106 (PMC8933735; doi:10.1001/jamanetworkopen.2022.2106)
Supplement: Supplement 2. — eMethods. eTable 1. Medical and Psychiatric History of the Overall Study Sample eTable 2. Concomitant Medications at Enrollment by Intervention Group eTable 3. Self-Reported CBD:THC Ratio and Method of Use by Intervention Group eTable 4. Cannabinoid Metabolite Measurements at Week 12 by Intervention Group eTable 5. Secondary Outcomes (SF-12 Mental and Physical Well-Being, and Cognitive Outcomes) by Intervention Group eTable 6. CUD Diagnosis by Group and Study Timepoint eTable 7. Cannabis Use Disorder Symptoms Endorsed in Each Group eTable 8. Exploratory Cannabis Use and Symptom Rating Outcomes eTable 9. Sample Characteristics at Screening Those Randomized to MMC Who Obtained vs Did Not Obtain MMC eTable 10. Sensitivity Analysis of Primary Outcomes by Randomization Group With Adjustment Using Propensity Scores eTable 11. Sensitivity Analysis of Primary Outcomes in MMC Participants With Metabolites, and in WLC Participants Without Detectable Metabolites eTable 12. Primary Outcomes in Participants With Blinded Assessments eTable 13. Treatment Emergent Adverse Events eTable 14. Severity of Treatment Emergent Adverse Events eFigure. CUD Symptoms by Intervention Group at Each Study Timepoint eReferences [file jamanetwopen-e222106-s002.pdf]

Supplementary Online Content

Gilman JM, Schuster RM, Potter KW, et al. Effect of medical marijuana card ownership on pain, insomnia, and affective disorder symptoms in adults: a randomized clinical trial. *JAMA Netw Open*. 2022;5(3):e222106. doi:10.1001/jamanetworkopen.2022.2106

eMethods.
eTable 1. Medical and Psychiatric History of the Overall Study Sample
eTable 2. Concomitant Medications at Enrollment by Intervention Group
eTable 3. Self-Reported CBD:THC Ratio and Method of Use by Intervention Group
eTable 4. Cannabinoid Metabolite Measurements at Week 12 by Intervention Group
eTable 5. Secondary Outcomes (SF-12 Mental and Physical Well-Being, and Cognitive Outcomes) by Intervention Group
eTable 6. CUD Diagnosis by Group and Study Timepoint
eTable 7. Cannabis Use Disorder Symptoms Endorsed in Each Group
eTable 8. Exploratory Cannabis Use and Symptom Rating Outcomes
eTable 9. Sample Characteristics at Screening Those Randomized to MMC Who Obtained vs Did Not Obtain MMC
eTable 10. Sensitivity Analysis of Primary Outcomes by Randomization Group With Adjustment Using Propensity Scores
eTable 11. Sensitivity Analysis of Primary Outcomes in MMC Participants With Metabolites, and in WLC Participants Without Detectable Metabolites
eTable 12. Primary Outcomes in Participants With Blinded Assessments
eTable 13. Treatment Emergent Adverse Events
eTable 14. Severity of Treatment Emergent Adverse Events
eFigure. CUD Symptoms by Intervention Group at Each Study Timepoint
eReferences

This supplementary material has been provided by the authors to give readers additional information about their work.

© 2022 Gilman JM et al. *JAMA Network Open.*

eMethods.

Inclusion Criteria:
1. Men and women aged 18-65 years, inclusive;
2. Competent and willing to provide written informed consent;
3. Desire to use medical marijuana for self-reported pain, sleep, or affective (mood and/or anxiety including PTSD) symptoms.
4. Not in possession of a medical marijuana card, but expressing intent to get one.
5. Able to communicate in English language.

Exclusion Criteria:
1. Current daily marijuana use (prior to enrollment).
2. Current substance use disorders (e.g. cocaine, opiate, stimulant, cannabis). Light to moderate alcohol use is permitted (defined as 16 or less on the AUDIT), and nicotine dependence is permitted because of the high co-use of nicotine and marijuana. Participants cannot meet current SCID criteria for a use disorder on any illicit substance.
3. Pregnant (verified by a urine test).
4. Diagnosis of acute coronary syndrome, coronary thrombosis, myocardial infarction, cardiomyopathies and arrhythmias such as atrial fibrillation, ventricular tachycardia or fibrillation, thrombophilia such as Factor V Leiden, Prothrombin 20210, antithrombin deficiency, antiphospholipid syndrome.
5. History of deep vein thrombosis (DVT), pulmonary embolism, stroke.
6. In the opinion of the investigator, not able to safely participate in this study because of any medical or psychological issues* that might compromise their safety, e.g. psychosis.

*Participants were excluded if they had evidence for any psychotic illness or were considered by the investigators to be at high risk of self-injury or suicidal behavior, as assessed by participants' responses on the Concise Health Risk Tracking scale (CHRT) or Mini International Neuropsychiatric Interview (MINI) suicidality module administered at screening, and, if necessary, professional mental health assessment.

Blinding. In our original study design, outcome assessors were blind to group assignment. Due to the coronavirus pandemic and the transition from in-person to virtual (video) visits in March 2020, when study nurse SH and physician GP was redeployed, scheduling difficulties made it necessary for study staff who had assessed baseline eligibility to complete some subsequent virtual assessments, thus were not assured to be blind to intervention allocation. This occurred for 19% of visits. Eighty-one percent of assessments were conducted by blinded raters. Sensitivity analyses conducted using only fully blinded assessments yielded similar results and are presented in eTable12.

CANTAB Battery

Participants completed 5 tasks from the Cambridge Neuropsychological Test Automated Battery (CANTAB).[1]

Attention Switching Task (AST): The AST has, on each trial, an arrow appear on the right or left hand side of the screen, and the participant is asked to make a right or left response. Preceding each trial, a cue is given indicating whether a participant should make a right or left response based on a) the direction of the arrow (irrespective of the side it appears on), or b) the position of the arrow (irrespective of the direction the arrow points). Trials can be congruent (the direction of the arrow and side it appears on are equivalent), or incongruent (the direction of the arrow differs from the side it appears on). Trials are also grouped into blocks, where some blocks involve a single type of cue (all trials are only direction-based or side-based), or both cues (trials can alternate between direction-

© 2022 Gilman JM et al. *JAMA Network Open.*

based and side-based cues). The key outcomes used in the analysis were 1) the difference in mean response times for all incongruent trials versus all congruent trials (lower values indicate greater adaptability in handling incongruent stimuli), and 2) the difference in mean response times for all trials from multi-cue blocks versus all trials from single-cue blocks (lower values indicate greater ability to multitask and adapt to different cues).

Rapid Visual Information Processing (RVP) task: The RVP task is a continuous performance task in which a participant views a sequence of digits (at a rate of 100 digits per minute). Participants must detect a target sequence of digits (e.g., 3-5-7); once they see the final digit of the sequence, they must make a response. The key outcome used in the analysis was a transformation of a) the hit rate (correct identification of target sequences) and b) the mean and standard deviation for response time for hits. These three measures were converted into a single measure of discriminability using the EZ-diffusion model.[2]

Paired Associates Learning (PAL) task: The PAL task has boxes displayed on screen that open one by one in a randomized order to reveal patterns hidden inside. The patterns are then displayed in the middle of the screen one at a time. The participant must touch the box in which a given pattern was originally located. If a participant makes an error, patterns are shown again to remind the participant of their locations. The key outcome used for analysis was the total number of errors committed (when a participant chose the wrong box for a given pattern) for all completed errors, plus an adjustment for estimated number of errors a participant would have made for trials that were not completed. Higher scores correspond with worse memory performance.

Spatial Working Memory (SWM) task: The SWM task starts with colored boxes shown on screen. A subset of boxes contain tokens - the participant opens the boxes and uses a process of elimination to identify which of the boxes contain these tokens. The key outcome used for analysis was the number of times a participant reopens a box he or she had already determined contained a token. Higher scores indicate worse spatial memory performance.

Verbal Recognition Memory (VRM): The VRM task consisted of 3 phases. First, participants are shown a list of words to study and commit to memory. Then, participants completed a free recall phase where they must list out loud all words they remember being in the studied list. Finally, participants are given a forced choice task where a word (either a studied word or a distractor item) is shown and the participant must indicate whether the word was studied or not. Key outcomes included in the analysis were a) the number of words correctly recalled, and b) d’ (a transformation of target words correctly identified as studied and distractor words incorrectly identified as studied into a measure of discriminability).

© 2022 Gilman JM et al. *JAMA Network Open.*

eTable 1. Medical and Psychiatric History of the Overall Study Sample

**Condition**	**MMC (n=105)**	**WLC (n=81)**	**Total (n=186)**
**Musculoskeletal Disorders**			
Head trauma	21 (20.0%)	17 (20.9%)	38 (20.4%)
Arthritis	18 (17.1%)	6 (7.4%)	24 (12.9%)
Back pain	5 (4.8%)	6 (7.4%)	11 (5.9%)
Concussion	4 (3.8%)	4 (4.9%)	8 (4.3%)
Herniated disc	4 (3.8%)	2 (2.5%)	6 (3.2%)
Osteoporosis	3 (2.9%)	2 (2.5%)	5 (2.7%)
**Neurological Disorders**			
Migraines	44 (41.9%)	27 (33.3%)	71 (38.2%)
Vertigo	20 (19.1%)	16 (19.8%)	36 (19.4%)
Fibromyalgia	3 (2.9%)	2 (2.5%)	5 (2.7%)
Headaches	2 (1.9%)	3 (3.7%)	5 (2.7%)
**Gastrointestinal Disorders**			
GERD	32 (30.5%)	24 (29.6%)	56 (30.1%)
Constipation	13 (12.4%)	5 (6.2%)	18 (9.7%)
IBS	9 (8.6%)	8 (9.9%)	17 (9.1%)
Gastritis	5 (4.8%)	2 (2.5%)	7 (3.8%)
**Cardiovascular Disorders**			
Hypercholesterolemia	11 (10.5%)	13 (16.1%)	24 (12.9%)
Hypo/hypertension	10 (9.5%)	12 (14.1%)	22 (11.8%)
Heart murmur	6 (5.8%)	2 (2.5%)	8 (4.3%)
Angina	5 (4.8%)	2 (2.5%)	7 (3.8%)
Arrhythmia	5 (4.8%)	2 (2.5%)	7 (3.8%)
**Respiratory Disorders**			
Asthma	24 (22.9%)	20 (24.7%)	44 (23.7%)
Bronchitis	5 (4.8%)	2 (2.5%)	7 (3.8%)
Chronic sinusitis	4 (3.8\%)	2 (2.5%)	6 (3.2%)
**Endocrine & Metabolic Disorders**			
Thyroid disease	8 (7.6%)	4 (4.9%)	12 (6.5%)
Hormone replacement	8 (7.6%)	3 (3.7%)	11 (5.9%)
Diabetes	5 (4.8%)	2 (2.5%)	7 (3.8%)
**Other Disorders**			
Anemia	15 (14.29%)	5 (6.2%)	20 (10.9%)
Cancer	8 (7.62%)	1 (1.2%)	9 (4.8%)
Appendicitis	4 (3.81%)	1 (1.2%)	5 (2.69%)
Psychiatric Disorders			
**Major Depressive Disorder**			
Current	16 (15.24%)	12 (14.81%)	28 (15.05%)
Past	64 (60.95%)	46 (56.79%)	110 (59.14%)
**Bipolar Disorder II (Past)**	1 (0.95%)	4 (4.94%)	5 (2.69%)
**Panic Disorder**			
Current	3 (2.86%)	4 (4.94%)	7 (3.76%)
Past	8 (7.62%)	8 (9.88%)	16 (8.6%)

© 2022 Gilman JM et al. *JAMA Network Open.*

Condition	MMC	WLC	Total
**Agoraphobia**	3 (2.86%)	2 (2.47%)	5 (2.69%)
**Social Anxiety**	9 (8.57%)	8 (9.88%)	17 (9.14%)
**Generalized Anxiety Disorder**	23 (21.9%)	16 (19.75%)	39 (20.97%)
**Post-Traumatic Stress Disorder**	4 (3.81%)	6 (7.41%)	10 (5.38%)
**Insomnia**	29 (27.88%)	21 (25.93%)	50 (27.03%)
**Somatic Symptom Disorder**	12 (11.54%)	7 (8.64%)	19 (10.27%)
**Alcohol Use Disorder**			
Current	5 (4.76%)	1 (1.23%)	6 (3.23%)
Past	22 (45.83%)	17 (44.74%)	39 (45.35%)
**Cannabis Use Disorder (Past)**	5 (8.06%)	10 (16.95%)	15 (12.4%)
**No MINI Diagnosis**	17 (16.19%)	14 (17.28%)	31 (16.67%)

Medical conditions were self-reported by the participant during a standard review of systems by a study physician, nurse, or other study staff. Psychological diagnosis were assessed via the MINI interview.
[a] Due to modifications in the MINI assessment throughout the study, sample size is smaller for the following groups: Alcohol Use Disorder (Past) (n=86); Past Substance Use Disorders (n=120); and Insomnia (Current), and Somatic Symptom Disorder (Current) (n=182). Conditions endorsed by less than 5 subjects are not reported.

© 2022 Gilman JM et al. *JAMA Network Open.*

eTable 2. Concomitant Medications at Enrollment by Intervention Group

Category	MMC (n=105)<br>n (%)	WLC (n=81)<br>n (%)	Total (n=186)<br>n (%)
Antidepressants	49 (46.7%)	35 (43.2%)	84 (45.2%)
NSAIDs	46 (43.8%)	38 (46.9%)	84 (45.2%)
Vitamins & Supplements	52 (49.5%)	25 (30.9%)	77 (41.4%)
Contraception/hormones	30 (28.6%)	20 (24.7%)	50 (26.9%)
Non-Opioid/Non-NSAID Pain Medication	19 (18.1%)	11 (13.6%)	30 (16.1%)
Antihypertensives	15 (14.3%)	13 (16%)	28 (15.1%)
CNS Stimulants	17 (16.2%)	9 (11.1%)	26 (14%)
Corticosteroid	16 (15.2%)	10 (12.3%)	26 (14%)
Antihistamine	14 (13.3%)	10 (12.3%)	24 (12.9%)
Bronchodilator/Leukotriene Inhibitors	14 (13.3%)	6 (7.4%)	20 (10.8%)
PPI/Antacids	8 (7.6%)	9 (11.1%)	17 (9.1%)
Muscle Relaxants	12 (11.4%)	3 (3.7%)	15 (8.1%)
Opioids	9 (8.6%)	6 (7.4%)	15 (8.1%)
Antihyperlipidemic	8 (7.6%)	5 (6.2%)	13 (7%)
Mood Stabilizers	6 (5.7%)	5 (6.2%)	11 (5.9%)
Thyroid Hormone	6 (5.7%)	4 (4.9%)	10 (5.4%)
Antibiotic	3 (2.9%)	6 (7.4%)	9 (4.8%)
Antidiabetic agents	4 (3.8%)	4 (4.9%)	8 (4.3%)
Triptan	3 (2.9%)	5 (6.2%)	8 (4.3%)
Immunosuppressant Agents	5 (4.8%)	1 (1.2%)	6 (3.2%)
Antiviral	2 (1.9%)	3 (3.7%)	5 (2.7%)
Antifungal	3 (2.9%)	2 (2.5%)	5 (2.7%)
Decongestant	3 (2.9%)	2 (2.5%)	5 (2.7%)
No Medications [a]	3 (2.9%)	6 (7.4%)	9 (4.8%)

Medications were self-reported by participants at time of enrollment. Medications endorsed by less than 5 subjects are not reported.
[a] When excluding contraception/hormones and vitamins & supplements, no medications = 7 (6.7%) in MMC, 5 (6.2%) in WLC, and 12 (6.5%) overall.

© 2022 Gilman JM et al. *JAMA Network Open.*

eTable 3. Self-Reported CBD:THC Ratio and Method of Use by Intervention Group

**Self-Report**	**Total, n (%)**	
	**MMC (n=100)**	**WLC (n=74)**
Self-Reported Product Types [a]		
CBD Dominant	11 (11.0%)	5 (6.8%)
Equal CBD and THC	17 (17.0%)	8 (10.8%)
THC Dominant	44 (44.0%)	18 (24.3%)
Unknown/Data Not Provided	21 (21.0%)	14 (18.9%)
Self-Reported Route of Administration [b]		
Oral	31 (31.0%)	14 (18.9%)
Smoked	18 (18.0%)	21 (28.4%)
Vaped	44 (44.0%)	10 (13.5%)
No Use Reported	7 (7.0%)	29 (39.2%)

[a] Participants were asked to report whether they believed their primary cannabis products were THC-dominant, CBD-dominant, or a balanced 'hybrid' product.
[b] Participants were asked to report the primary method via which they consumed cannabis.
Please note, 12-week data were only available for 100 in MMC and 74 in WLC groups.

© 2022 Gilman JM et al. *JAMA Network Open.*

eTable 4. Cannabinoid Metabolite Measurements at Week 12 by Intervention Group

Variable	Limit of Quantification (ng/mL)	Participants with detected metabolites No. (%)	
THC Metabolites		MMC (n=100)	WLC (n=74)
11OH-THC	1.56-400	0 (0%)	1 (1.4%)
THC	0.78-400	0 (0%)	0 (0.0%)
THC-COOH	0.39-400	31 (31%)	17 (23.0%)
THC-COO-Gluc	7.8-2000	55 (55%)	23 (31.1%)
THC-Gluc	0.78-400	10 (10%)	8 (10.8%)
THCV	0.78-400	0 (0.0%)	0 (0.0%)
THCV-COOH [a]	0.78-400	4 (4.0%)	3 (4.1%)
CBD Metabolites			
6a-OH-CBD	0.78-400	2 (2.0%)	2 (2.7%)
6b-OH-CBD	0.78-400	1 (1.0%)	2 (2.7%)
7-CBD-COOH	0.78-400	4 (4.0%)	12 (16.2%)
7-OH-CBD	0.78-400	2 (2.0%)	5 (6.8%)
CBD	0.78-400	0 (0.0%)	1 (1.4%)
CBD-Gluc	0.78-100	31 (31.0%)	8 (10.8%)
CBDV	0.39-400	0 (0.0%)	0 (0.0%)
Any THC Metabolite		55 (55.0%)	24 (32.4%)
Any CBD Metabolite		34 (34.0%)	15 (20.3%)
Any Metabolite		59 (59.0%)	26 (35.1%)

Urine samples were stored at -80C until they were shipped overnight on dry ice to the University of Colorado School of Medicine for quantitative metabolite assay that measured 17 different cannabis metabolites, according to established procedures validated in human urine, in which an online extraction, high-performance liquid chromatography coupled with tandem mass spectrometry method was performed on each sample. Only those metabolites that were detected are listed. None of the following metabolites were detected in any sample: CBC, CBG, CBN, THC, and THCV. Please note, 12-week data were only available for 100 in MMC and 74 in WLC groups.

Abbreviations: THC-COO-Gluc =11-nor-Δ9-tetrahydrocannabinol-9-carboxylic acid glucuronide; THC-COOH = 11-Nor-9-carboxy-Δ9-tetrahydrocannabinol; THC-Gluc = Δ9-tetrahydrocannabinol glucuronide; THCV-COOH = 11-nor-9-carboxy- Δ9-tetrahydrocannabivarin; 1-OH-THC = 11-hydroxy- Δ9-tetrahydrocannabinol; HC = Δ9-tetrahydrocannabinol; THCV = Δ9-tetrahydrocannabivarin; CBD-Gluc = Cannabidiol glucuronide; 7-CBD-COOH = (3R-trans)-Cannabidiol-7-oic Acid; 7-OH-CBD = 7-hydroxy-cannabidiol; 6α-OH-CBD = 6-alpha-hydroxy-cannabidiol; 6β-OH-CBD = 6-beta-hydroxy-cannabidiol; CBD = Cannabidiol; CBDV = Cannabidivarin; CBC = Cannabichromene; CBG = Cannabigerol; CBN = Cannabinol

[a] THCV-COOH only has values for 106 samples due to lack of reference THCV-COOH in 1 batch. 36 non-uses' samples were not shipped and imputed as the lower limit of quantification (LLOQ).

© 2022 Gilman JM et al. *JAMA Network Open.*

eTable 5. Secondary Outcomes (SF-12 Mental and Physical Well-Being, and Cognitive Outcomes) by Intervention Group

Outcome	Visit	Mean (SD); N MMC	Mean (SD); N WLC	Cohen's d (95% CI)
Mental well-being [a]	Baseline	41.0 (12.4); 104	42.3 (11.3); 78	
	Week 4	44.7 (11.5); 101	40.6 (12.1); 76	
	Week 12	45.7 (11.0); 100	41.9 (13.0); 74	
Est. difference	MMC vs. WLC	4.7 (2.6 to 6.7)		0.39 (0.21 to 0.58)
Physical well-being [a]	Baseline	46.2 (11.8); 104	48.4 (11.4); 78	
	Week 4	46.6 (11.1); 101	48.0 (12.1); 76	
	Week 12	47.7 (10.5); 100	48.3 (11.9); 74	
Est. difference	MMC vs. WLC	0.8 (-1.0 to 2.5)		0.07 (-0.10 to 0.22)
Congruency cost (reaction time) [b]	Baseline	64.9 (49.7); 95	81.1 (52.5); 67	
	Week 4	60.6 (39.9); 95	78.8 (50.0); 67	
	Week 12	65.3 (45.6); 86	75.0 (49.4); 59	
Est. difference	MMC vs. WLC	-7.7 (-19.0 to 3.5)		-0.15 (-0.38 to 0.07)
Switching cost (reaction time) [b]	Baseline	218.5 (124.8); 95	217.9 (108.5); 67	
	Week 4	150.1 (91.7); 95	176.2 (105.4); 67	
	Week 12	127.5 (87.3); 86	146.8 (107.7); 59	
Est. difference	MMC vs. WLC	-24.1 (-45.9 to -2.2)		-0.20 (-0.38 to -0.02)
Discriminability [c]	Baseline	0.05 (0.04); 101	0.03 (0.03); 76	
	Week 4	0.07 (0.05); 99	0.06 (0.04); 75	
	Week 12	0.08 (0.05); 92	0.06 (0.05); 70	
Est. difference	MMC vs. WLC	0.00 (-0.01 to 0.01)		0.06 (-0.19 to 0.29)
Total errors [d]	Baseline	12.6 (12.3); 101	12.7 (13.4); 76	
	Week 4	9.7 (11.7); 99	9.9 (11.6); 75	
	Week 12	8.5 (10.9); 92	8.6 (11.5); 70	
Est. difference	MMC vs. WLC	-0.1 (-1.8 to 1.7)		0.00 (-0.16 to 0.13)
Repetition errors [e]	Baseline	33.0 (20.1); 101	32.8 (19.3); 76	
	Week 4	26.6 (18.4); 99	30.9 (20.6); 75	
	Week 12	26.2 (18.5); 92	29.0 (19.0); 69	
Est. difference	MMC vs. WLC	-3.6 (-7.4 to 0.3)		-0.18 (-0.37 to 0.03)
d' (recognition memory) [f]	Baseline	2.54 (0.77); 98	2.58 (0.67); 73	
	Week 4	2.54 (0.79); 96	2.63 (0.64); 71	
	Week 12	2.62 (0.72); 90	2.73 (0.71); 66	
Est. difference	MMC vs. WLC	-0.08 (-0.24 to 0.08)		-0.11 (-0.33 to 0.11)
Total correct (Free recall) [f]	Baseline	7.7 (2.8); 101	7.7 (2.9); 74	
	Week 4	8.7 (2.5); 99	8.0 (2.7); 73	
	Week 12	9.2 (2.9); 93	9.0 (2.8); 67	
Est. difference	MMC vs. WLC	0.4 (-0.2 to 1.0)		0.15 (-0.07 to 0.38)

Estimated difference and associated effect sizes are based on general estimating equations linear model.

© 2022 Gilman JM et al. *JAMA Network Open.*

[a] The 12-item Short-Form Health Surveys (SF-12 Physical and SF-12 Mental) has a range of 0-100 and is scored using a z-score, rescaled to have a mean of 50 (SD=10), with higher scores indicating better health status.
[b] Congruency and switching cost were assessed with the Attention Switching Task (AST), which assesses attention shifting and executive function. The AST was not able to be administered remotely after the transition to virtual visits in March 2020; therefore, data is missing in 42 (8%) of visits where CANTAB was conducted virtually.
[c] Discriminability was assessed with the Rapid Visual Information Processing (RVP) task, which assesses sustained attention.
[d] Total errors were assessed with the Paired Associates Learning (PAL), which assesses visual memory.
[e] Repetition errors were assessed with the Spatial Working Memory (SWM) task, which assesses spatial working memory and executive function.
[f] d' and total recall were assessed with the Verbal Recognition Memory task (VRM), which assesses verbal memory.

© 2022 Gilman JM et al. *JAMA Network Open.*

eTable 6. CUD Diagnosis by Group and Study Timepoint

Group	Week	N	Mild CUD	Moderate CUD
MM	2	101	6 (5.9%)	1 (1%)
	4	102	8 (7.8%)	0 (0%)
	12	100	8 (8%)	2 (2%)
WLC	2	80	1 (1.2%)	1 (1.2%)
	4	78	1 (1.3%)	0 (0%)
	12	74	4 (5.4%)	0 (0%)

Reported percentage is out of total in each group. Please note, there were no incidences of severe CUD in any participant.

eTable 7. Cannabis Use Disorder Symptoms Endorsed in Each Group

Cannabis Use Disorder Symptom	Times Endorsed	
	MMC	WLC
01: The substance is often taken in larger amounts OR over a longer period than was intended.	4	0
02: There is a persistent desire OR unsuccessful efforts to cut down or control substance use.	6	0
03: A great deal of time is spent in activities necessary to obtain the substance, use the substance, or recover from its effects.	3	1
04: Craving, or a strong desire or urge to use the substance.	4	3
05: Recurrent substance use resulting in a failure to fulfill major role obligations at work, school, or home.	0	0
06: Continued substance use despite having persistent or recurrent social or interpersonal problems caused or exacerbated by the effects of the substance.	0	0
07: Important social, occupational, or recreational activities given up or reduced because of substance use.	1	0
08: Recurrent substance use in situations in which it is physically hazardous.	6	0
09: Substance use is continued despite knowledge of having a persistent or recurrent physical or psychological problem that is likely to have been caused or exacerbated by the substance.	12	2
10: Tolerance, as defined by: a. A need for markedly increased amounts of the substance to achieve intoxication or desired effect. b. Markedly diminished effect with continued use of the same amount of the substance.	15	3
11: Withdrawal, as manifested by either of the following: a. The characteristic withdrawal syndrome for the substance. b. The same (or a closely related) substance is taken to relieve or avoid withdrawal symptoms.	4	3

© 2022 Gilman JM et al. *JAMA Network Open.*

eTable 8. Exploratory Cannabis Use and Symptom Rating Outcomes

Outcome	Visit	Mean (SD); N		Cohen's d
		MMC	WLC	(95% CI)
Cannabis use disorder [a]	Baseline	2.79 (2.58); 105	3.09 (3.08); 81	
	Week 2	4.88 (2.80); 101	3.22 (2.96); 80	
	Week 4	5.33 (2.65); 102	3.09 (3.06); 78	
	Week 12	5.48 (2.93); 100	3.43 (3.34); 74	
Est. difference	MMC vs. WLC	2.22 (1.65 to 2.78)		0.8 (0.6 to 1.0)
Marijuana craving [b]	Baseline	10.47 (4.56); 77	9.57 (4.28); 56	
	Week 2	9.14 (3.71); 74	9.00 (3.50); 56	
	Week 4	8.60 (4.18); 75	8.06 (3.59); 51	
	Week 12	8.47 (4.69); 72	8.68 (4.53); 50	
Est. difference	MMC vs. WLC	-0.27 (-1.16 to 0.61)		-0.1 (-0.3 to 0.1)
Pain interference [c]	Baseline	2.59 (2.40); 37	3.52 (2.59); 24	
	Week 2	2.63 (2.12); 37	3.77 (2.66); 24	
	Week 4	2.21 (2.30); 36	3.20 (2.92); 22	
	Week 12	2.32 (2.35); 37	3.22 (3.17); 21	
Est. difference	MMC vs. WLC	-0.59 (-1.61 to 0.44)		-0.2 (-0.7 to 0.2)
Pain catastrophizing [d]	Baseline	11.62 (9.38); 37	13.88 (10.00); 24	
	Week 2	10.95 (8.92); 37	13.83 (10.68); 24	
	Week 4	10.14 (7.47); 36	11.64 (9.56); 22	
	Week 12	8.08 (6.68); 37	14.67 (14.66); 21	
Est. difference	MMC vs. WLC	-2.15 (-5.25 to 0.95)		-0.2 (-0.6 to 0.1)
Perceived stress [e]	Baseline	16.65 (7.30); 105	17.00 (7.46); 81	
	Week 2	15.48 (7.19); 101	17.55 (7.96); 80	
	Week 4	14.89 (7.13); 102	17.31 (7.70); 78	
	Week 12	14.53 (6.53); 100	17.27 (7.52); 74	
Est. difference	MMC vs. WLC	-2.09 (-3.19 to -0.99)		-0.3 (-0.4 to -0.1)
Suicidal thoughts [f]	Baseline	20.50 (6.77); 40	19.28 (7.01); 32	
	Week 2	19.78 (7.15); 37	18.94 (7.05); 32	
	Week 4	18.16 (6.37); 38	18.48 (6.71); 31	
	Week 12	18.78 (6.39); 36	18.64 (6.96); 28	
Est. difference	MMC vs. WLC	-0.81 (-2.45 to 0.83)		-0.1 (-0.4 to 0.1)
CGI Illness severity [g]	Baseline	3.31 (1.17); 105	3.30 (1.20); 81	
	Week 2	3.12 (1.20); 101	3.33 (1.09); 80	
	Week 4	3.10 (1.21); 101	3.12 (1.15); 78	
	Week 12	3.00 (1.27); 100	2.93 (1.20); 74	
Est. difference	MMC vs. WLC	-0.07 (-0.29 to 0.15)		-0.1 (-0.3 to 0.1)
CGI Global improvement [g]	Baseline	0.06 (0.62); 105	-0.04 (0.62); 81	
	Week 2	-0.19 (0.85); 101	0.05 (0.74); 80	
	Week 4	-0.47 (0.94); 101	0.06 (0.86); 78	
	Week 12	-0.49 (1.12); 100	-0.15 (1.09); 74	

© 2022 Gilman JM et al. *JAMA Network Open.*

Outcome	Visit	Mean (SD); N		Cohen's d
		MMC	WLC	(95% CI)
Est. difference	MMC vs. WLC	-0.37 (-0.56 to -0.19)		-0.6 (-0.9 to -0.3)

Estimated difference and associated effect sizes are based on general estimating equations linear model.

[a] Cannabis Use Disorders Identification Test score range is 0-32. Higher scores indicated more problematic use. Scores of 8 or more indicate hazardous cannabis use, while scores of 12 or more indicate a possible cannabis use disorder for which further intervention may be required.

[b] Marijuana Craving Questionnaire score range is 12-84. Higher scores indicate more craving, and a score of 84 would indicate that a participant strongly agreed with all items.

[c] Brief Pain Inventory Pain Interference score range is 0-10, with 10 being complete interference. This measure was analyzed only in participants with a primary complaint of pain.

[d] Pain Catastrophizing Scale score range is 0-52. Higher scores indicate more catastrophizing. This measure was analyzed only in participants with a primary complaint of pain.

[e] Perceived Stress Scale score range is 0-40. Higher scores indicate more stress. A score of 0-13 are considered low stress, 14-26 is considered moderate stress, and 27-40 is considered high perceived stress. This measure was analyzed in all participants.

[f] Concise Health Risk Tracking Scale range is 12-60. Higher scores indicate more suicidal ideation, and a score of 60 would indicate that a participant strongly agreed with all items. This measure was analyzed only in participants with a primary complaint of depression or anxiety.

[g] Clinician Global Impression of Change scale. The illness severity scale range is 1-7, where higher scores indicate greater severity. The improvement scale range is 1-7, where higher scores indicate worsening of the condition compared to baseline. This measure was analyzed in all participants.

© 2022 Gilman JM et al. *JAMA Network Open.*

eTable 9. Sample Characteristics at Screening Those Randomized to MMC Who Obtained vs Did Not Obtain MMC

Measure	Obtained MMC	Did not obtain MMC
Sample size	120	53
Age; M (SD)	37.1 (14.2)	38.2 (14.3)
Female; n (%)	81 (67.5%)	37 (69.8%)
Race; n (%)		
Caucasian	99 (82.5%)	33 (62.3%)
Non-Caucasian	21 (17.5%)	20 (37.7%)
Hispanic; n (%)	6 (5%)	6 (11.3%)
Education years; M (SD)	16.6 (2.6)	15.8 (2.8)
Cannabis Use Frequency ≥ weekly; n (%)	27 (22.5%)	14 (26.4%)
Primary complaint [a]; n (%)		
Pain	39 (32.5%)	26 (49.1%)
Insomnia	24 (20%)	9 (17%)
Anxiety/depression	57 (47.5%)	18 (34%)
Primary outcomes; M (SD)		
Pain severity [b]	1.7 (2.1)	2.5 (2.6)
Insomnia symptoms [c]	1.7 (2.1)	2.5 (2.6)
Depression symptoms [d]	4.9 (3.4)	5.2 (4.4)
Anxiety symptoms [d]	8 (4.4)	7.8 (4.5)

[a] Primary complaint was defined by participant self-report of the condition for which they were seeking medical cannabis.

[b] Symptoms of pain were assessed via the severity subscale of the Brief Pain Inventory (BPI; 0-10, 10 being worst imaginable pain). This measure was analyzed only in participants with a primary complaint of pain.

[c] Symptoms of insomnia were assessed via the Athens Insomnia Scale (AIS; 0-24 scale; higher scores indicate more acute sleep difficulties). This measure was analyzed only in participants with a primary complaint of insomnia.

[d] Symptoms of depression and anxiety were assessed by the Hospital Anxiety and Depression Scale (HADS; 0-21 scale for anxiety and depression separately; with higher scores indicating worsening anxiety or depression; 0-7:normal, 8-10: borderline abnormal (borderline case); 11-21 abnormal (case)). This measure was analyzed only in participants with a primary complaint of depression or anxiety.

© 2022 Gilman JM et al. *JAMA Network Open.*

eTable 10. Sensitivity Analysis of Primary Outcomes by Randomization Group With Adjustment Using Propensity Scores

Outcome	Visit	Mean (SD); N		Cohen's d
		MMC	WLC	
CUD symptoms [a]	Baseline	0.08 (0.27); 105	0.09 (0.28); 81	
	Week 2	0.30 (0.67); 101	0.16 (0.56); 80	
	Week 4	0.33 (0.68); 102	0.05 (0.36); 78	
	Week 12	0.55 (0.95); 100	0.16 (0.50); 74	
Est. difference	MMC vs. WLC	0.28 (0.14 to 0.42)		1.01 (0.53 to 1.59)
Pain severity [b]	Baseline	2.8 (2.3); 37	3.9 (2.4); 24	
	Week 2	3.2 (2.2); 37	3.6 (2.4); 24	
	Week 4	2.4 (2.3); 36	3.1 (2.6); 22	
	Week 12	2.5 (2.4); 37	3.1 (2.6); 21	
Est. difference	MMC vs. WLC	0.1 (-0.7 to 1.0)		0.04 (-0.36 to 0.40)
Insomnia	Baseline	12.4 (4.4); 22	12.2 (2.7); 20	
	Week 2	10.0 (5.4); 21	11.6 (3.7); 19	
	Week 4	8.8 (3.4); 22	12.1 (2.6); 20	
	Week 12	7.6 (4.9); 22	11.2 (4.7); 20	
Est. difference	MMC vs. WLC	-2.9 (-4.3 to -1.6)		-0.80 (-1.30 to -0.44)
Depression Symptoms	Baseline	6.1 (3.7); 46	5.2 (4.3); 37	
	Week 2	6.0 (4.5); 43	4.4 (3.6); 37	
	Week 4	5.2 (4.1); 44	5.2 (3.9); 36	
	Week 12	4.9 (4.1); 41	5.5 (4.2); 33	
Est. difference	MMC vs. WLC	-0.3 (-1.3 to 0.7)		-0.08 (-0.34 to 0.18)
Anxiety Symptoms	Baseline	9.4 (4.4); 46	9.4 (4.1); 37	
	Week 2	8.3 (4.3); 43	8.3 (3.8); 37	
	Week 4	8.5 (4.5); 44	8.4 (4.1); 36	
	Week 12	8.3 (4.4); 41	8.4 (3.7); 33	
Est. difference	MMC vs. WLC	-0.1 (-1.1 to 0.9)		-0.02 (-0.30 to 0.23)

Estimated raw and adjusted difference and associated effect sizes are based on general estimating equations linear model.

[a] Symptoms of CUD were assessed by the CUD Checklist for DSM-5 (0-11 scale; higher scores indicate more severe CUD). This measure was analyzed in all participants.

[b] Symptoms of pain were assessed via the severity subscale of the Brief Pain Inventory (BPI; 0-10, 10 being worst imaginable pain). This measure was analyzed only in participants with a primary complaint of pain.

[c] Symptoms of insomnia were assessed via the Athens Insomnia Scale (AIS; 0-24 scale; higher scores indicate more acute sleep difficulties). This measure was analyzed only in participants with a primary complaint of insomnia.

[d] Symptoms of depression and anxiety were assessed by the Hospital Anxiety and Depression Scale (HADS; 0-21 scale for anxiety and depression separately; with higher scores indicating worsening anxiety or depression; 0-7:normal, 8-10: borderline abnormal (borderline case); 11-21 abnormal (case)). This measure was analyzed only in participants with a primary complaint of depression or anxiety.

© 2022 Gilman JM et al. *JAMA Network Open.*

eTable 11. Sensitivity Analysis of Primary Outcomes in MMC Participants With Metabolites, and in WLC Participants Without Detectable Metabolites

Outcome	Visit	Mean (SD); N		Cohen's d
		MMC	WLC	
CUD symptoms [a]	Baseline	0.09 (0.29); 86	0.09 (0.29); 68	
	Week 2	0.44 (0.82); 54	0.08 (0.33); 62	
	Week 4	0.43 (0.76); 63	0.00 (0.00); 53	
	Week 12	0.63 (0.89); 59	0.06 (0.32); 48	
Est. difference	MMC vs. WLC	0.44 (0.30 to 0.58)		1.52 (1.05 to 2.19)
Pain severity [b]	Baseline	3.0 (2.3); 32	3.8 (2.6); 19	
	Week 2	2.9 (2.3); 21	3.6 (2.4); 19	
	Week 4	2.8 (2.3); 28	2.9 (2.9); 17	
	Week 12	3.0 (2.6); 21	3.0 (2.8); 16	
Est. difference	MMC vs. WLC	0.2 (-0.9 to 1.2)		0.07 (-0.39 to 0.48)
Insomnia	Baseline	12.8 (4.8); 16	11.9 (2.7); 17	
	Week 2	11.6 (4.1); 8	11.8 (4.2); 14	
	Week 4	8.6 (4.0); 13	12.2 (2.9); 14	
	Week 12	7.8 (5.5); 13	12.4 (5.2); 11	
Est. difference	MMC vs. WLC	-3.9 (-5.5 to -2.2)		-1.01 (-1.59 to -0.52)
Depression Symptoms	Baseline	6.5 (3.7); 38	5.2 (4.3); 32	
	Week 2	5.5 (4.4); 25	4.6 (3.7); 29	
	Week 4	4.7 (4.7); 22	6.0 (3.9); 22	
	Week 12	5.1 (4.4); 25	6.0 (4.6); 21	
Est. difference	MMC vs. WLC	-0.8 (-1.9 to 0.3)		-0.20 (-0.49 to 0.06)
Anxiety Symptoms	Baseline	9.9 (4.3); 38	9.5 (4.2); 32	
	Week 2	7.9 (4.8); 25	8.6 (3.9); 29	
	Week 4	6.9 (4.2); 22	9.0 (4.3); 22	
	Week 12	8.5 (4.8); 25	8.3 (3.8); 21	
Est. difference	MMC vs. WLC	-0.3 (-1.5 to 1.0)		-0.06 (-0.35 to 0.22)

Estimated raw and adjusted difference and associated effect sizes are based on general estimating equations linear model. This analysis included only those in the MMC group with detectable THC or CBD metabolites at Week 12, and those in the WLC group with no detectable metabolites at Week 12.

[a] Symptoms of CUD were assessed by the CUD Checklist for DSM-5 (0-11 scale; higher scores indicate more severe CUD). This measure was analyzed in all participants.

[b] Symptoms of pain were assessed via the severity subscale of the Brief Pain Inventory (BPI; 0-10, 10 being worst imaginable pain). This measure was analyzed only in participants with a primary complaint of pain.

[c] Symptoms of insomnia were assessed via the Athens Insomnia Scale (AIS; 0-24 scale; higher scores indicate more acute sleep difficulties). This measure was analyzed only in participants with a primary complaint of insomnia.

[d] Symptoms of depression and anxiety were assessed by the Hospital Anxiety and Depression Scale (HADS; 0-21 scale for anxiety and depression separately; with higher scores indicating worsening anxiety or depression; 0-7:normal, 8-10: borderline abnormal (borderline case); 11-21 abnormal (case)). This measure was analyzed only in participants with a primary complaint of depression or anxiety.

© 2022 Gilman JM et al. *JAMA Network Open.*

eTable 12. Primary Outcomes in Participants With Blinded Assessments

Outcome	Visit	Mean (SD); N		Cohen's d	p-value
		MMC	WLC	(95% CI)	
CUD symptoms [a]	Baseline	0.06 (0.24); 99	0.10 (0.30); 73		
	Week 2	0.29 (0.71); 56	0.31 (0.77); 39		
	Week 4	0.33 (0.71); 81	0.07 (0.41); 59		
	Week 12	0.57 (0.97); 74	0.21 (0.55); 58		
Est. difference	MMC vs. WLC	0.27 (0.10 to 0.44)		1.02 (0.38 to 1.78)	p = 0.001
Pain severity [b]	Baseline	2.8 (2.3); 37	3.9 (2.4); 22		
	Week 2	2.6 (2.1); 19	3.9 (2.8); 12		
	Week 4	2.6 (2.3); 30	3.3 (2.7); 18		
	Week 12	2.9 (2.5); 28	2.6 (2.4); 17		
Est. difference	MMC vs. WLC	0.2 (-0.7 to 1.1)		0.07 (-0.31 to 0.45)	p = 0.709
Insomnia symptoms [c]	Baseline	12.2 (4.4); 21	12.1 (2.9); 17		
	Week 2	10.9 (3.5); 13	11.0 (2.7); 8		
	Week 4	8.7 (3.7); 19	11.7 (2.5); 16		
	Week 12	7.6 (4.5); 15	9.9 (4.6); 14		
Est. difference	MMC vs. WLC	-2.2 (-3.7 to -0.7)		-0.59 (-1.14 to -0.19)	p = 0.004
Depression symptoms [d]	Baseline	6.1 (3.8); 41	5.5 (4.3); 34		
	Week 2	5.5 (4.5); 24	4.8 (4.0); 19		
	Week 4	4.8 (4.4); 32	5.2 (3.3); 25		
	Week 12	4.9 (4.2); 31	5.8 (4.2); 27		
Est. difference	MMC vs. WLC	-0.8 (-1.9 to 0.3)		-0.20 (-0.48 to 0.08)	p = 0.137
Anxiety symptoms [d]	Baseline	9.2 (4.5); 41	9.7 (4.1); 34		
	Week 2	7.6 (4.6); 24	8.7 (3.8); 19		
	Week 4	8.1 (4.5); 32	8.8 (3.3); 25		
	Week 12	8.7 (4.4); 31	8.9 (3.7); 27		
Est. difference	MMC vs. WLC	-0.3 (-1.5 to 0.9)		-0.07 (-0.35 to 0.21)	p = 0.634

Estimated difference and associated effect sizes and p-value based on general estimating equations linear model.
[a] Symptoms of CUD were assessed by the CUD Checklist for DSM-5 (0-11 scale; higher scores indicate more severe CUD). This measure was analyzed in all participants.

[b] Symptoms of pain were assessed via the severity subscale of the Brief Pain Inventory (BPI; 0-10, 10 being worst imaginable pain). This measure was analyzed only in participants with a primary complaint of pain.

[c] Symptoms of insomnia were assessed via the Athens Insomnia Scale (AIS; 0-24 scale; higher scores indicate more acute sleep difficulties). This measure was analyzed only in participants with a primary complaint of insomnia.

[d] Symptoms of depression and anxiety were assessed by the Hospital Anxiety and Depression Scale (HADS; 0-21 scale for anxiety and depression separately; with higher scores indicating worsening anxiety or depression; 0-7:normal, 8-10: borderline abnormal (borderline case); 11-21 abnormal (case)). This measure was analyzed only in participants with a primary complaint of depression or anxiety.

© 2022 Gilman JM et al. *JAMA Network Open.*

eTable 13. Treatment Emergent Adverse Events

Category	MMC n=105	WLC n=81
**Infections and infestations**	**50 (47.6%)**	**31 (38.3%)**
Nasopharyngitis	38 (36.2%)	24 (29.6%)
**Psychiatric symptoms**	**31 (29.5%)**	**21 (25.9%)**
Worsened Anxiety	18 (17.1%)	10 (12.3%)
Worsened Stress	10 (9.5%)	8 (9.9%)
Worsened Depressed mood	10 (9.5%)	9 (11.1%)
**General disorders and conditions**	**32 (30.5%)**	**13 (16%)**
Worsened Pain	15 (14.3%)	8 (9.9%)
Fatigue	6 (5.7%)	1 (1.2%)
**Nervous system symptoms**	**13 (12.7%)**	**8 (9.9%)**
Dizziness	6 (5.7%)	3 (3.7%)
Headache	3 (2.9%)	3 (3.7%)
**Injury and poisoning**	**12 (11.4%)**	**10 (12.3%)**
Joint injury	6 (5.7%)	2 (2.5%)
Muscle injury	2 (1.9%)	4 (4.9%)
**Gastrointestinal disorders**	**14 (13.3%)**	**5 (6.2%)**
Nausea	9 (8.6%)	3 (3.7%)
**Respiratory, thoracic and mediastinal disorders**	**7 (6.9%)**	**4 (4.9%)**
Oropharyngeal Pain	5 (4.8%)	1 (1.2%)
**Musculoskeletal disorders**	**2 (1.9%)**	**8 (9.9%)**
**Immune system disorders**	**4 (3.8%)**	**5 (6.2%)**
**No AEs reported**	**15 (14.3%)**	**21 (25.9%)**

Reported percentage is out of total in each group. Main row for each group is based on MedDRA SOC Terms while the sub-row is based on the MedDRA PT Term. Only sub-categories or main categories that contained 5 or more adverse events are displayed. Other for main categories included: Blood and lymphatic system disorders; Cardiac disorders; Ear and labyrinth disorders; Endocrine disorders; Eye disorders; Hepatobiliary disorders; Metabolism and nutrition disorders; Neoplasms benign, malignant and unspecified (incl cysts and polyps); Renal and urinary disorders; Reproductive system and breast disorders; Skin and subcutaneous tissue disorders; Social circumstances; Surgical and medical procedures; and Vascular disorders.

© 2022 Gilman JM et al. *JAMA Network Open.*

eTable 14. Severity of Treatment Emergent Adverse Events

Category	Active n=105	WLC n=81
**Infections and infestations**	**50 (47.6%)**	**31 (38.3%)**
Mild	21 (20%)	13 (16%)
Moderate	37 (35.2%)	21 (25.9%)
Severe	0 (0.0%)	1 (1.2%)
**Psychiatric disorders**	**31 (29.5%)**	**21 (25.9%)**
Mild	15 (14.3%)	12 (14.8%)
Moderate	13 (12.4%)	11 (13.6%)
Severe	9 (8.6%)	5 (6.2%)
**General disorders and conditions**	**32 (30.5%)**	**13 (16%)**
Mild	8 (7.6%)	2 (2.5%)
Moderate	23 (21.9%)	12 (14.8%)
Severe	3 (2.9%)	1 (1.2%)
**Nervous system disorders**	**17 (16.2%)**	**8 (9.9%)**
Mild	6 (5.7%)	4 (4.9%)
Moderate	10 (9.5%)	3 (3.7%)
Severe	1 (1%)	1 (1.2%)
**Injury, poisoning and complications**	**12 (11.4%)**	**10 (12.3%)**
Mild	2 (1.9%)	1 (1.2%)
Moderate	10 (9.5%)	9 (11.1%)
Severe	0 (0.0%)	0 (0.0%)
**Gastrointestinal disorders**	**14 (13.3%)**	**5 (6.2%)**
Mild	5 (4.8%)	3 (3.7%)
Moderate	8 (7.6%)	1 (1.2%)
Severe	1 (1%)	1 (1.2%)
**Respiratory disorders**	**8 (7.6%)**	**5 (6.2%)**
Mild	5 (4.8%)	3 (3.7%)
Moderate	3 (2.9%)	2 (2.5%)
Severe	0 (0.0%)	0 (0.0%)
**Musculoskeletal disorders**	**2 (1.9%)**	**8 (9.9%)**
Mild	1 (1%)	2 (2.5%)
Moderate	1 (1%)	6 (7.4%)
Severe	0 (0.0%)	0 (0.0%)
**Immune system disorders**	**4 (3.8%)**	**5 (6.2%)**
Mild	3 (2.9%)	3 (3.7%)

© 2022 Gilman JM et al. *JAMA Network Open.*

Category	Active	WLC
Moderate	1 (1%)	2 (2.5%)
Severe	0 (0.0%)	0 (0.0%)

Reported percentage is out of total in each group. Main row for each group is MedDRA's SOC Term while the sub-row is Severity. Only sub-categories or main categories that contained 5 or more adverse events in any group are displayed.

© 2022 Gilman JM et al. *JAMA Network Open.*

eFigure. CUD Symptoms by Intervention Group at Each Study Timepoint

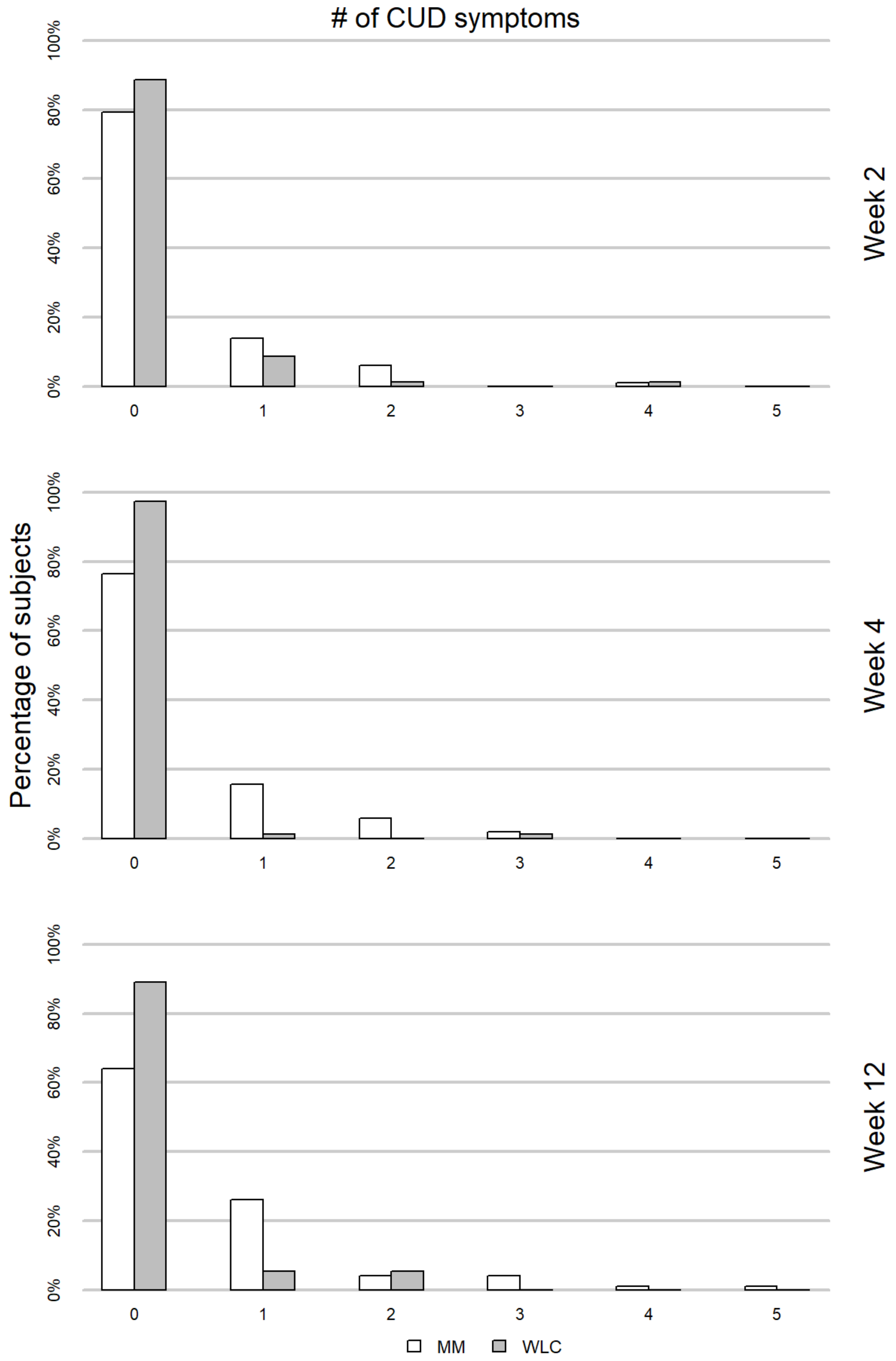

© 2022 Gilman JM et al. *JAMA Network Open.*

eReferences

1. Robbins TW, James M, Owen AM, Sahakian BJ, McInnes L, Rabbitt P. Cambridge Neuropsychological Test Automated Battery (CANTAB): a factor analytic study of a large sample of normal elderly volunteers. *Dementia.* 1994;5(5):266-281.
2. Wagenmakers EJ, van der Maas HL, Grasman RP. An EZ-diffusion model for response time and accuracy. *Psychon Bull Rev.* 2007;14(1):3-22.

© 2022 Gilman JM et al. *JAMA Network Open.*
