# Supplementary material for: Effect of Medical Marijuana Card Ownership on Pain, Insomnia, and Affective Disorder Symptoms in Adults: A Randomized Clinical Trial
Source: JAMA Netw Open. 2022 Mar 18;5(3):e222106. doi: 10.1001/jamanetworkopen.2022.2106 (PMC8933735; doi:10.1001/jamanetworkopen.2022.2106)
Supplement: Supplement 3. — Data Sharing Statement [file jamanetwopen-e222106-s003.pdf]

Data Sharing Statement

Gilman. Effect of Medical Marijuana Card Ownership on Pain, Insomnia, and Affective Disorder Symptoms in Adults. *JAMA Netw Open*. Published March 18, 2022.
doi:10.1001/jamanetworkopen.2022.2106

Data
Data available: Yes
Data types: Deidentified participant data, Data dictionary
How to access data: All data, code, and materials used in the analyses can be provided by Jodi Gilman and Massachusetts General Hospital pending scientific review and a completed data use agreement/material transfer agreement. Requests for all materials should be submitted to Jodi Gilman at jgilman1@mgh.harvard.edu.
When available: beginning date: 09-01-2022

Supporting Documents
Document types: Statistical/analytic code, Informed consent form
How to access documents: All materials can be provided by Jodi Gilman and Massachusetts General Hospital pending scientific review and a completed data use agreement/material transfer agreement. Requests for all materials should be submitted to Jodi Gilman at jgilman1@mgh.harvard.edu.
When available: With publication

Additional Information
Who can access the data: All data, code, and materials used in the analyses can be provided by Jodi Gilman and Massachusetts General Hospital pending scientific review and a completed data use agreement/material transfer agreement.
Types of analyses: All data, code, and materials used in the analyses can be provided by Jodi Gilman and Massachusetts General Hospital for scientific purpose pending scientific review.
Mechanisms of data availability: All materials used in the analyses can be provided via a completed data use agreement/material transfer agreement.
